# Anti-cancer effects of curcumin on lung cancer through the inhibition of EZH2 and NOTCH1

**DOI:** 10.18632/oncotarget.8532

**Published:** 2016-04-01

**Authors:** Guo-Qing Wu, Ke-Qun Chai, Xiu-Ming Zhu, Hua Jiang, Xiao Wang, Qian Xue, Ai-Hong Zheng, Hong-Ying Zhou, Yun Chen, Xiao-Chen Chen, Jian-Yong Xiao, Xu-Hua Ying, Fu-Wei Wang, Tao Rui, Yi-Ji Liao, Dan Xie, Li-Qin Lu, Dong-Sheng Huang

**Affiliations:** ^1^ Department of Oncology and Cancer Biotherapy Center, Zhejiang Provincial People's Hospital, Hangzhou, Zhejiang 310014, China; ^2^ Zhejiang Academy of Traditional Chinese Medicine, Tongde Hospital of Zhejiang Province, Hangzhou, Zhejiang 310012, China; ^3^ Department of Biochemistry, Guangzhou University of Chinese Medicine, Guangzhou 510006, China; ^4^ State Key Laboratory of Oncology in South China, Cancer Center, Sun Yat-Sen University, Guangzhou 510060, Guangdong, China

**Keywords:** curcumin, enhancer of zeste homolog 2, NOTCH1, lung cancer, microRNA

## Abstract

Curcumin is potentially therapeutic for malignant diseases. The mechanisms of this effect might involve a combination of antioxidant, immunomodulatory, proapoptotic, and antiangiogenic activities. However, the exact mechanisms are not fully understood. In the present study, we provided evidences that curcumin suppressed the expression of enhancer of zeste homolog 2 (EZH2) in lung cancer cells both transcriptionally and post-transcriptionally. Curcumin inhibited the expression of EZH2 through microRNA (miR)-let 7c and miR-101. Curcumin decreased the expression of NOTCH1 through the inhibition of EZH2. There was a reciprocal regulation between EZH2 and NOTCH1 in lung cancer cells. These observations suggest that curcumin inhibits lung cancer growth and metastasis at least partly through the inhibition of EZH2 and NOTCH1.

## INTRODUCTION

Lung cancer is the most commonly diagnosed cancer and is the leading cause of cancer death in males globally [1]. Although much progress has been made in the past 10 years for lung cancer [2, 3], even in developed countries such as the United States of America, only 16.8% of all lung cancer patients survive for 5 years or more after diagnosis [4]. Therefore, a better understanding of the mechanism of lung cancer development and new options for its treatment are warranted.

Curcumin, a component of turmeric, was reported to have anti-cancer effects and is a potential agent for the prevention and treatment of malignant diseases including lung cancer. Curcumin blocks cell transformation, proliferation, and invasion and induces cell apoptosis [5]. Several studies over the past three decades have established curcumin's involvement in several biochemical pathways. The molecular targets of curcumin include growth factors [6], growth factor receptors [7], transcription factors [8, 9], cytokines [10, 11], enzymes [8, 12], and genes regulating apoptosis and proliferation [13, 14].

We were the first to report that the enhancer of zeste homolog 2 (EZH2) is a target of curcumin [15]. EZH2, a polycomb group protein homolog to the *Drosophila* enhancer of zest, is a key component of the human polycomb repressive complex 2 that trimethylates histone H3K27 [16]. Previous studies from our and other laboratories have provided strong evidence in favor of EZH2′s oncogenic role. Overexpression of EZH2 was associated with tumor malignancy and a poor prognosis in human cancers including nasopharyngeal esophageal, breast, gastric, hepatic, pancreatic, ovarian, and bladder cancers [17–19]. Inhibition of EZH2 is a potential therapeutic approach for the treatment of malignant diseases [20, 21]. Here, we explored curcumin-mediated regulation of EZH2 and the underlying mechanism. Our investigation is the first to extensively explore the relationship between curcumin and EZH2 in lung cancer cells and the reciprocal regulation between EZH2 and NOTCH1.

## RESULTS

### Curcumin inhibits the proliferation, migration, invasion, and cell cycle progression of lung cancer cells

**Figure 1:**
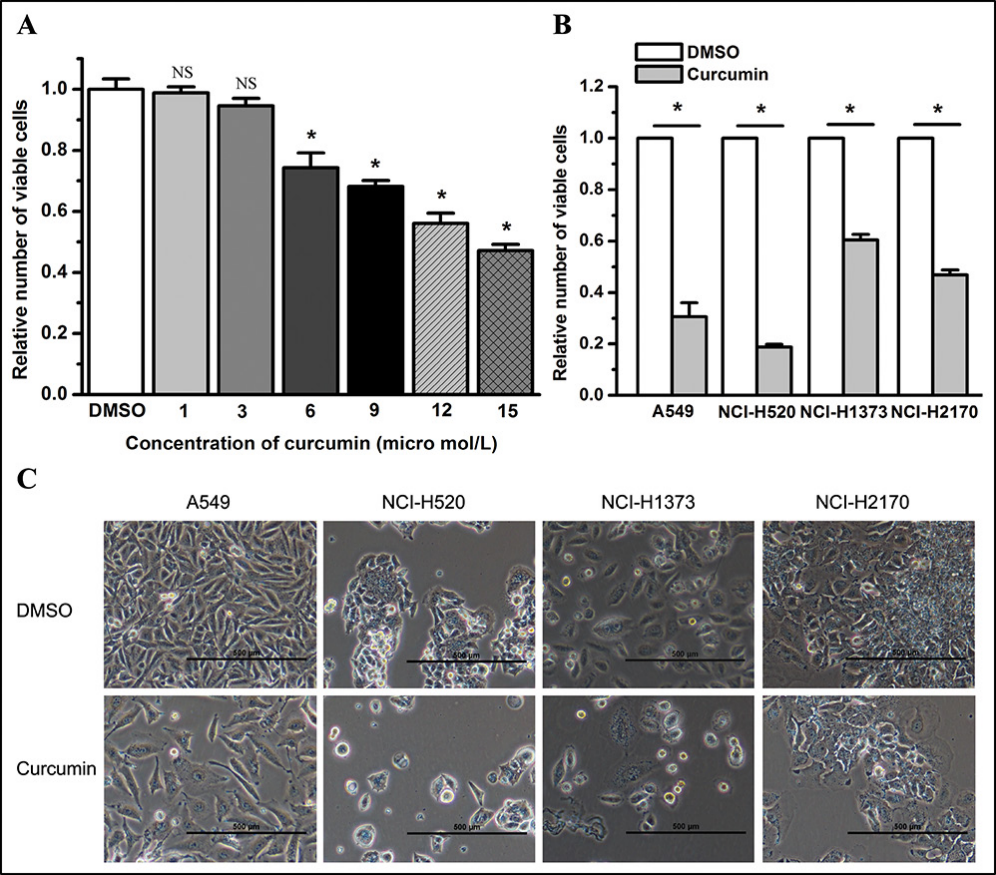
Curcumin inhibits the cell growth of lung cancer cells (**A**) Curcumin treatment (6 μM, 48 hours) inhibited the growth of A549 cells dose-dependently. NS, not statistically significant. *P < 0.05. (**B**) Curcumin treatment (6 μM, 72 hours) decreased the number of viable cancer cells as determined by the enumeration of viable cells. *P < 0.05. (**C**) Representative graphs for lung cancer cell lines A549, NCI-H520, NCI-H1373 and NCI-H2170 treated by 6 μM curcumin for 72 hours. Magnification bars = 500 μm. The viable cell number of the curcumin group was normalized to 1 for the DMSO group. All data shown represent the mean of at least three independent experiments. The data in all bar graphs are plotted as the mean ± SEM.

We examined the effect of curcumin on lung cancer cell proliferation by treating cells with curcumin at a final concentration of 1, 3, 6, 9, 12, or 15 μM. We found that curcumin dose-dependently inhibited the cell proliferation of lung cancer cell lines A549, NCI-H520, NCI-H1373, and NCI-H2170 at 48 hours post treatment (*P* < 0.05) (Figure 1A and data not shown). Compared to dimethylsulfoxide (DMSO), curcumin, at a final concentration of 6 μM, significantly inhibited the cell proliferation of lung cancer cells at 72 hours post treatment (*P* < 0.05) (Figure 1B and 1C).

**Figure 2:**
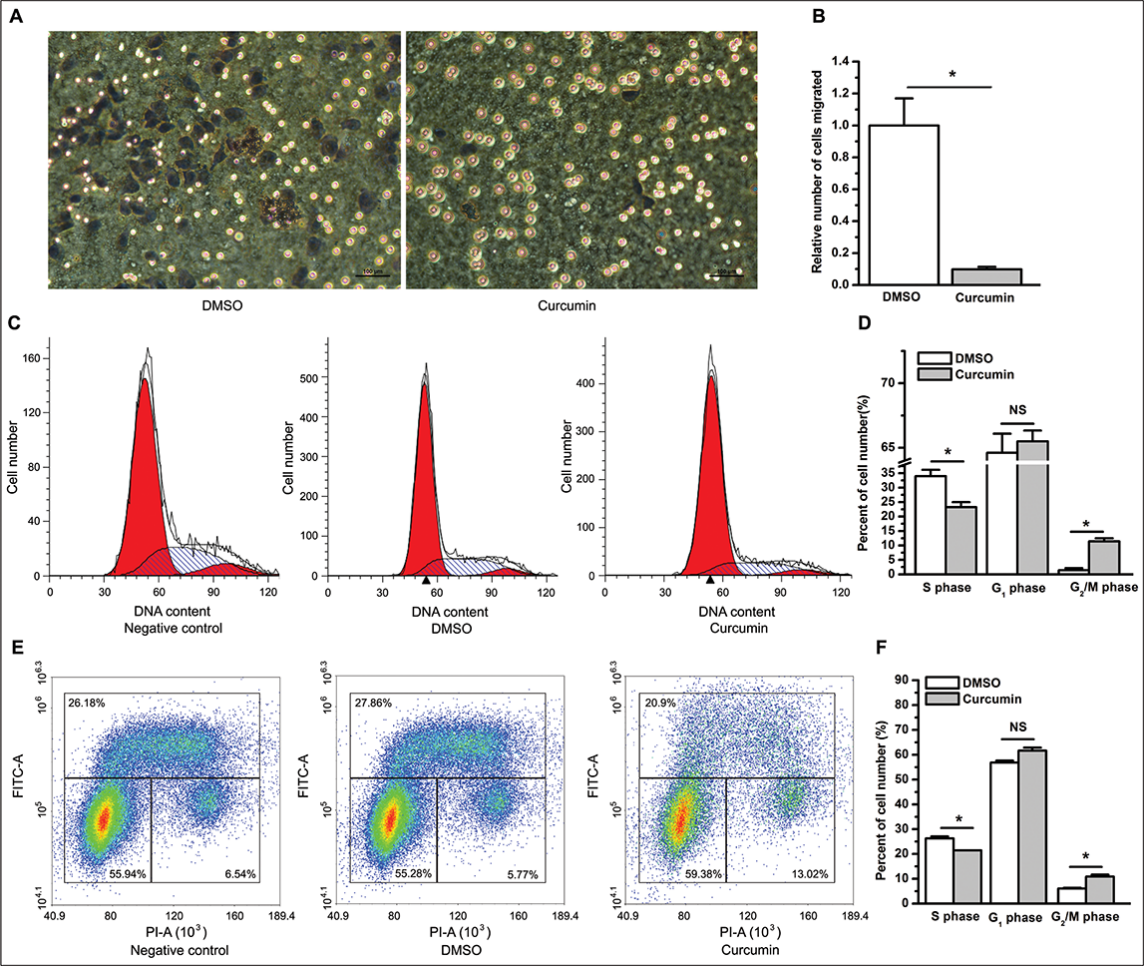
Curcumin suppresses cell migration and causes cell cycle arrest (**A**) Compared to the control treatment of DMSO (left), curcumin inhibited A549 cells from migrating through the membrane of transwell inserts (right). (**B**) The relative number of cells that migrated through the membrane of transwell inserts. *P < 0.05. The number of migrated cells in the curcumin group was normalized to 1 for the DMSO group. The cell migration assay was repeated three times with similar results, and triplicate inserts were included in each experiment. The data are plotted as the mean ± SEM. (**C**) Representative cell cycle distribution profiles obtained from A549 cells without treatment (left), treatment with DMSO (middle), and treatment with curcumin (right). (**D**) Curcumin decreased the percentage of cells in the S phase but increased the percentage of cells in the G_1_ (however, this increase was not statistically significant) and G_2_/M phases. *P < 0.05; NS, not statistically significant. (**E**) Representative cell cycle distribution profiles analyzed using BrdU-PI labeling for A549 cells without treatment (left), treatment with DMSO (middle), and treatment with curcumin (right). (**F**) BrdU-PI assay suggested that curcumin decreased the percentage of cells in the S phase, and increased the percentage of cells in the G_2_/M phase. *P < 0.05. The cell cycle analysis was performed three times with similar results, and triplicate flasks were included in each experiment. The data are plotted as the mean ± SEM.

Curcumin was previously reported to inhibit the cell migration and invasion of a variety of cancer cell lines *in vitro* [22, 23]. We further determined whether curcumin suppresses cell migration and invasion of lung cancer cells using a cell migration assay and a Matrigel invasion assay using transwell cell culture inserts and Matrigel invasion chambers, respectively. The results from the cell migration assay showed that compared with DMSO, curcumin significantly restrained lung cancer cells from migrating through the permeable transwell insert membrane at 9 hours post cell plating (*P* < 0.05) (Figure 2A and 2B). The Matrigel invasion assay suggested that compared to DMSO, curcumin significantly inhibited cell invasion through the Matrigel basement membrane matrix at 72 hours post cell plating (*P* < 0.05) (Supplementary Figure S1A, S1B). Because curcumin exerts an inhibitory effect on lung cancer cell proliferation, to rule out the possibility that the less number of viable cells trans-membraned in the curcumin group was the result of curcumin's suppressive effect on cell proliferation, we determined the number of viable cells incubated in medium with 1% or 10% FBS between the DMSO and the curcumin group at 9 hours and 72 hours post cell plating. As expected, the number of viable cells incubated in medium with 1% FBS was very similar at 9 hours post cell plating between the DMSO and the curcumin group (NS, not statistically significant, Supplementary Figure S1C). Similar results were found when using medium with 10% FBS (data not shown). These results suggest that the significant differences observed in the results from the cell migration assay were attributed to the inhibitorty effect of curcumin on cell migration. However, regardless of the concentration of FBS, the counts of viable cells from the curcumin group were much less than that from the DMSO group at 72 hours post cell plating (*P* < 0.05, Supplementary Figure S1D). This finding made it difficult to discern whether the significant differences of the results from the cell invasion assay between the DMSO and the curcumin group were the result of an inhibition of invasion, proliferation or both, which contributed to the suppressive results of curcumin on the cell invasion assay.

In addition to inhibiting cell proliferation, *in vitro* studies in various tumor cell lines demonstrated that curcumin causes cell cycle arrest and induces cell apoptosis [24, 25]. To determine whether curcumin affects cell cycle distribution and apoptosis of lung cancer cells, we used flow cytometry to examine cell cycle profiles and cell apoptosis after treatment with curcumin. Consistent with previous studies, compared to the control treatment of DMSO, curcumin significantly caused an arrest of A549 cells in the G_2_/M phase (*P* < 0.05), and decreased the percentage of cells in the S phase (*P* < 0.05). Although curcumin-treated cells appeared to show a higher percentage of cells in the G_1_ phase than DMSO-treated control cells, the result was not statistically significant (NS, not statistically significant. Figure 2C–2F).

In contrast to previous studies, staining with annexin V did not show significant increase of apoptosis in cells treated with curcumin compared to those treated with DMSO (NS, not statistically significant, Supplementary Figure S2). Concordantly, cell cycle analysis did not show a significant difference in the percentage of sub-G_0_ cells between cells treated with curcumin and DMSO (data not shown).

### Curcumin targets EZH2 in lung cancer cells

**Figure 3:**
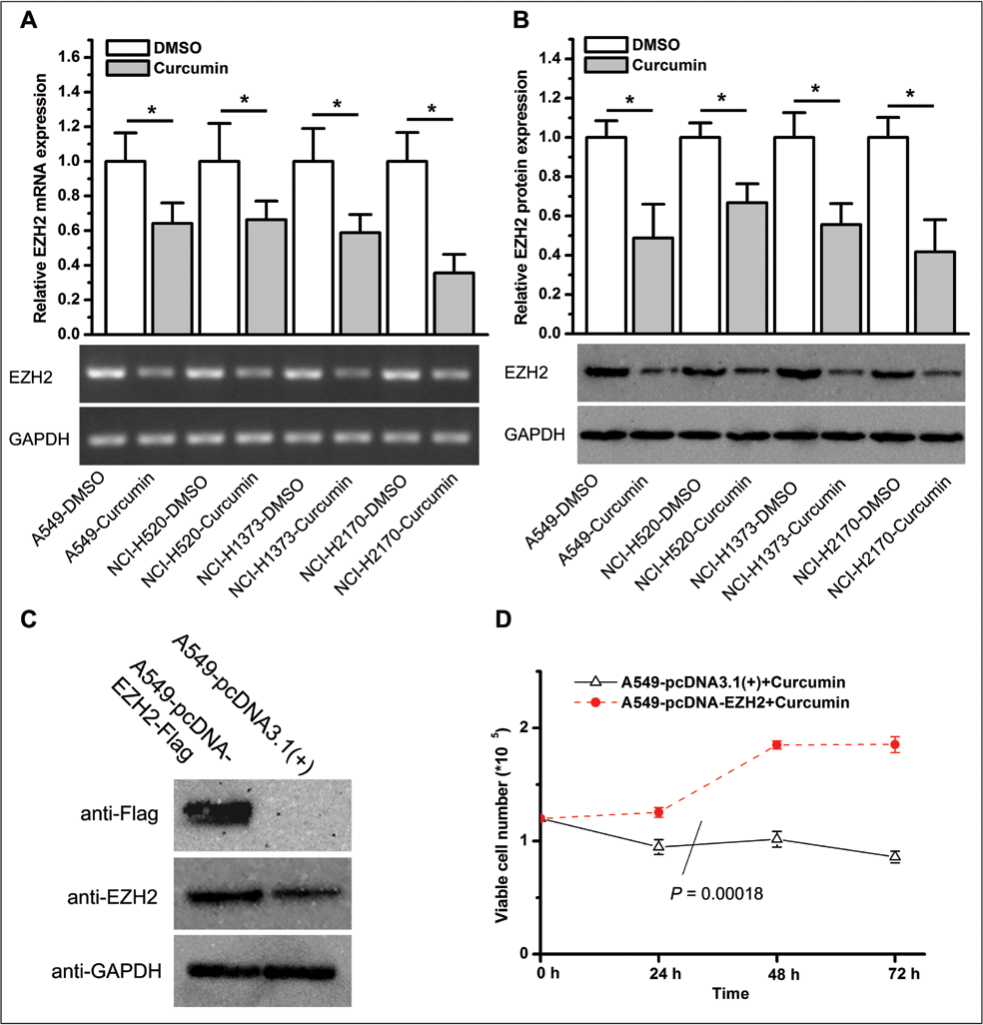
Curcumin inhibits EZH2 expression, and ectopic overexpression of EZH2 increases the resistance of lung cancer cells to curcumin (**A**) EZH2 mRNA expression in lung cancer cell lines treated with curcumin or DMSO for 72 hours. *P < 0.05. The bar graphs represent the actin beta (ACTB)-normalized EZH2 mRNA levels in lung cancer cell lines treated with curcumin relative to those treated with DMSO. (**B**) EZH2 protein expression in lung cancer cell lines treated with curcumin or DMSO for 72 hours. *P < 0.05. The bar graphs represent the glyceraldehyde-3-phosphate dehydrogenase (GAPDH)-normalized EZH2 protein levels in lung cancer cell lines treated with curcumin relative to those treated with DMSO. (**C**) Western blot analysis of flag-tagged EZH2 expression with anti-flag and anti-EZH2 antibodies in A549 cells. (**D**) Ectopic overexpression of EZH2 in A549 cells lead to increased tolerance to curcumin as determined by the enumeration of viable cells after the treatment of curcumin for 24 hours, 48 hours, and 72 hours. *P = 0.00018. All of the data shown represent the mean of at least three independent experiments and are plotted as the mean ± SEM.

Previous studies in our laboratory and others demonstrated that EZH2 was involved in tumor progression through the regulation of cell growth, apoptosis and invasion [17, 18, 26]. We have also reported that curcumin inhibited EZH2 expression in breast cancer cells [15]. To determine whether curcumin regulates EZH2 expression in lung cancer cells, we treated cell lines A549, NCI-H520, NCI-H1373, and NCI-H2170 with 6 μM of curcumin for 72 hours. qPCR and western blot analysis showed that curcumin significantly downregulated EZH2 mRNA and protein expression in lung cancer cells (Figure 3A and 3B).

Curcumin inhibits lung cancer cell proliferation and EZH2 expression, and several studies suggest that *ezh2* is a candidate oncogene. Thus, we speculate that curcumin's anti-cancer effects were due, at least in part, to the inhibition of EZH2. If this is indeed the case, forced overexpression of EZH2 is likely to increase the resistance of lung cancer cells to curcumin. To test this hypothesis, we established an A549 cell line that stably expressing exogenous EZH2 by transfecting cells with an *ezh2* plasmid followed by selection with selective media. Compared to A549 cells transfected with a control plasmid, cells stably transfected with an *ezh2* plasmid showed ectopic overexpression of flag-tagged EZH2 (Figure 3C) and were more resistant to curcumin (*P* = .00018, Figure 3D).

**Figure 4:**
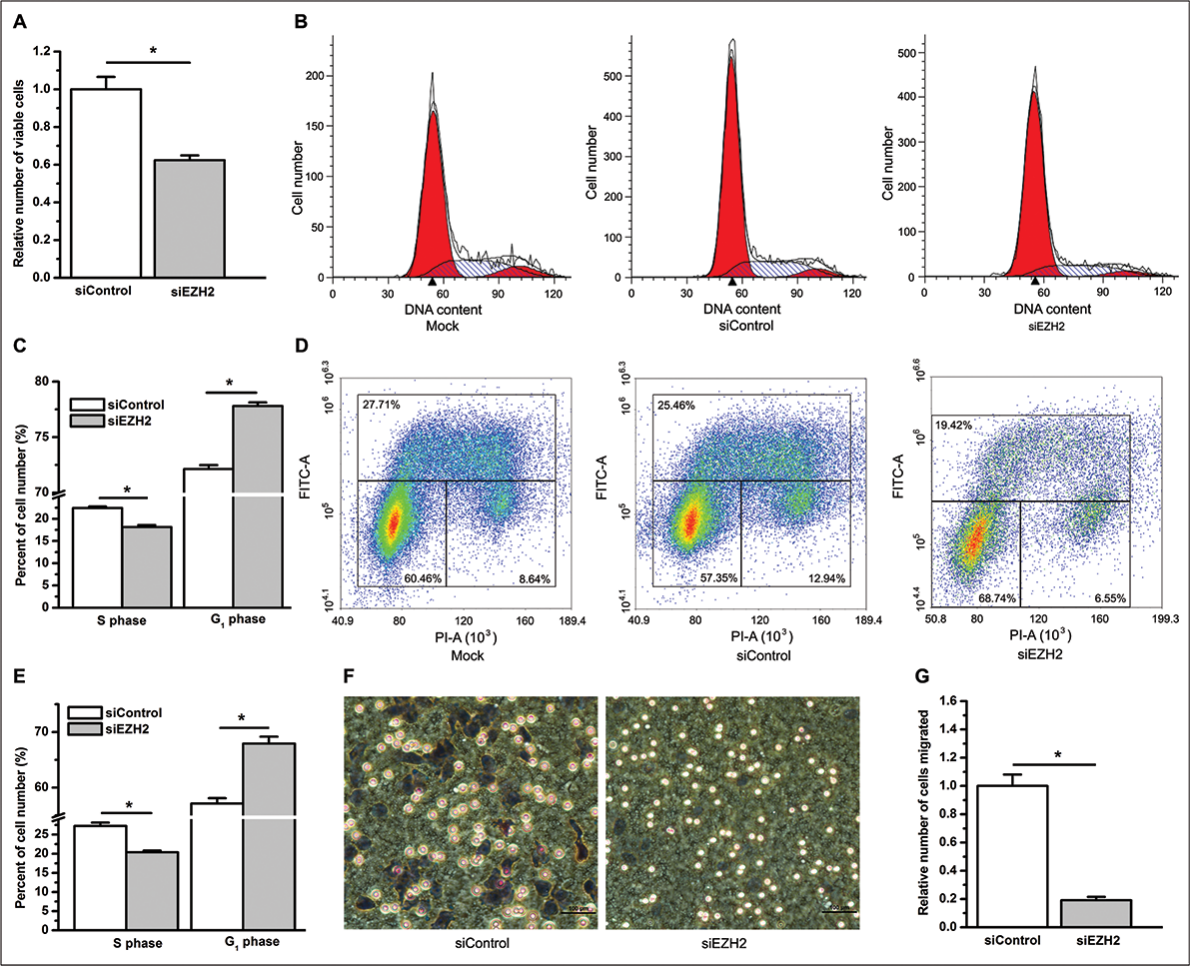
RNA interference knockdown of EZH2 decreases cell proliferation, induces cell cycle arrest, and suppresses cell migration (**A**) Knockdown of EZH2 by siRNA decreased the number of viable cells as determined by counting the number of viable cells. *P < 0.05. (**B**) Representative cell cycle distribution profiles obtained from A549 cells mock transfected using Lipofectamine (left), transfected with siControl (middle), and transfected with siEZH2 (right). (**C**) Knockdown of EZH2 with siRNA decreased the percentage of cells in the S phase and increased the percentage of cells in the G1 phase. *P < 0.05. (**D**) Representative cell cycle distribution profiles analyzed using BrdU-PI labeling for A549 cells mock transfected using Lipofectamine (left), transfected with siControl (middle), and transfected with siEZH2 (right). (**E**) BrdU-PI assay suggested that knockdown of EZH2 with siRNA decreased the percentage of cells in the S phase, and increased the percentage of cells in the G1 phase. *P < 0.05. (**F**) Compared to A549 cells transfected with siControl (left), A549 cells transfected with siEZH2 were suppressed from migrating through the membrane of transwell inserts (right). Magnification bars = 100 μm. (**G**) The relative number of cells that migrated through the membrane of transwell inserts. *P < 0.05. The number of viable A549 cells transfected with siEZH2 was normalized to 1 for that of viable A549 cells transfected with siControl. The cell cycle analysis was performed three times with similar results and triplicate flasks were included in each experiment. The number of migrated cells in the siEZH2 group was normalized to 1 for that of migrated cells in the siControl group. All of the data shown represent the mean of at least three independent experiments. The data in all bar graphs are plotted as the mean ± SEM.

Next, we knocked down EZH2 expression using a small interfering RNA (siRNA) (Supplementary Figure S3) and found that inhibition of EZH2 expression significantly suppressed cell growth, migration and G_1_ arrest of the cell cycle in A549 cells (*P* < 0.05, Figure 4A–4G). Because knockdown of EZH2 by siRNA against EZH2 (siEZH2) exerts an inhibitory effect on lung cancer cell proliferation, to rule out the possibility that the less number of viable cells trans-membraned in the siEZH2 group was the result of siEZH2′s suppressive effect on cell proliferation, we determined the number of viable cells incubated in medium with 1% or 10% FBS between the siRNA against a control (siControl) and the siEZH2 group at 9 hours post cell plating. Comparable to the data of curcumin, the number of viable cells incubated in medium with 1% FBS was very similar at 9 hours post cell plating between the siControl and the siEZH2 group (NS, not statistically significant, Supplementary Figure S4). Similar results were found when using medium with 10% FBS (data not shown). Nevertheless, neither the cell proliferation nor the cell cycle profile was changed by stable overexpression of EZH2 in A549 cells (Supplementary Figure S5).

**Figure 5:**
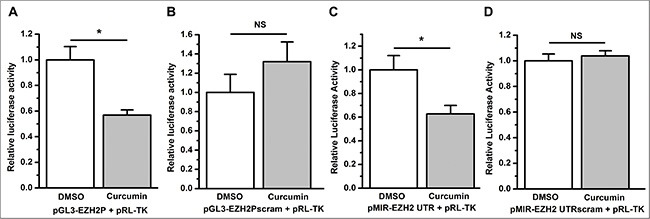
Curcumin inhibits the transcription and 3′ UTR stability of EZH2 (**A**) Curcumin significantly decreased firefly luciferase activity relative to control treatment of DMSO as determined by a dual luciferase reporter assay with A549 cells co-transfected with pGL3-EZH2P and the control vector pRL-TK. *P < 0.05. (**B**) Compared to the control treatment of DMSO, curcumin treatment did not change the luciferase activity, as determined by a luciferase reporter assay with A549 cells co-transfected with pGL3-EZH2Pscram and pRL-TK. NS, not statistically significant. (**C**) Curcumin significantly decreased luciferase activity relative to the control treatment of DMSO as determined by a luciferase reporter assay with A549 cells co-transfected with pMIR-EZH2 UTR and pRL-TK. *P < 0.05. (**D**) Compared to the control treatment of DMSO, curcumin did not change luciferase activity, as determined by a luciferase reporter assay with A549 cells co-transfected with pMIR-EZH2 UTRscram and pRL-TK. NS, not statistically significant. The luciferase activity (hluc:hRluc) was normalized to 1 for the control treatment of DMSO. The data in all bar graphs are plotted as the mean ± SEM.

To demonstrate whether curcumin regulates EZH2 expression at the transcriptional level or post-transcriptionally, we generated luciferase reporter vectors with the *ezh2* promoter or 3′ untranslated region (UTR) inserted. Compared to DMSO, curcumin significantly inhibited *ezh2* promoter activity in A549 cells transfected with an *ezh2* promoter luciferase reporter vector (*P* < 0.05, Figure 5A). This inhibition effect was completely abolished when A549 cells were transfected with the luciferase reporter vector harboring a sequence-scrambled *ezh2* promoter (NS, not statistically significant, Figure 5B), which suggests that curcumin inhibits the transcription of the *ezh2* gene.

When A549 cells were co-transfected with the EZH2 3′ UTR reporter vector pMIR-EZH2 UTR and the internal control vector pRL-TK, treatment with curcumin resulted in a significant reduction in luciferase activity compared with the control treatment of DMSO (*P* < 0.05, Figure 5C). Similarly, scrambling the 3′ UTR sequence of EZH2 on the luciferase reporter vector completely abrogated curcumin's regulatory activity (NS, not statistically significant, Figure 5D), thereby indicating that EZH2 expression is also post-transcriptionally regulated by curcumin.

### Curcumin downreguates EZH2 expression through miR-let 7c and miR-101

**Figure 6:**
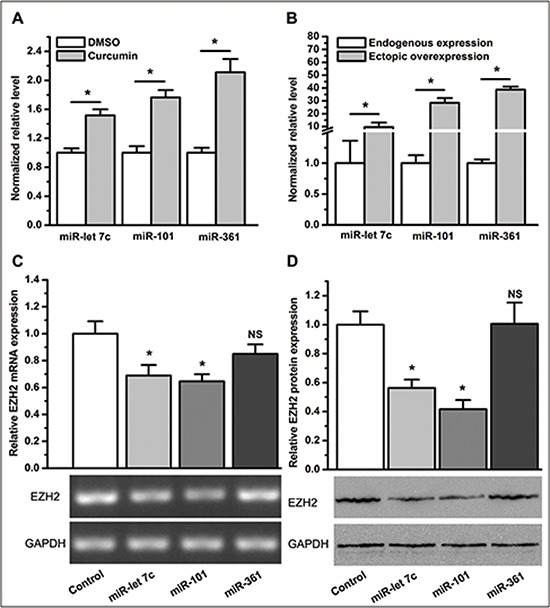
Curcumin changes the expression profile of miRNAs and the inhibitory effect of miR-let 7c and miR-101 on EZH2 expression (**A**) qPCR revealed that compared to DMSO, curcumin increased the expression level of miR-let 7c, miR-101, and miR-361. *P < 0.05. (**B**) Expression levels of miR-let 7c, miR-101, and miR-361 in A549 cells were significantly elevated after transduction with LV-miR-let 7c, LV-miR-101, and LV-miR-361, respectively. *P < 0.05. (**C**) Ectopic overexpression of miR-let 7c or miR-101, but not miR-361, lead to a lower expression level of EZH2 mRNA. *P < 0.05; NS, not statistically significant. (**D**) Ectopic overexpression of miR-let 7c or miR-101, but not miR-361, lead to a lower level of protein expression of EZH2. *P < 0.05; NS, not statistically significant. The expression level of each miRNA and EZH2 mRNA was normalized to U6 and ACTB, respectively. Protein expression levels of EZH2 were normalized to GAPDH. Expression levels of miRNAs were normalized to 1 for the control treatment of DMSO. Expression levels of miRNAs and EZH2 mRNA or protein were normalized to 1 for the control transduction of LV-control. The data are plotted as the mean ± SEM from three independent PCR amplifications or western blot assays.

Because the 3′ UTR of EZH2 is directly targeted by several miRNAs, we next sought to determine whether curcumin altered the expression of miRNA(s), which regulate EZH2 in lung cancer cells. For expression profiling of miRNAs in A549 cells treated with curcumin or control treated with DMSO, we conducted a miRNA array analysis and identified eight miRNAs (miR-let 7c, miR-101, miR-215, miR-361, miR-379–5p, miR-376a, miR-579, and miR-1247) with increased expression by ≥ 2-fold in cells treated with curcumin compared to those control treated with DMSO. To further validate the miRNA array result, we performed qPCR to quantify the relative expression level of the miRNAs mentioned above. Consistent with the result from the miRNA array, qPCR confirmed that curcumin significantly upregulated the expression of miR-let 7c, miR-101 and miR-361 (*P* < 0.05, Figure 6A). However, there was no statistically significant difference for the remaining five miRNAs between A549 cells treated with curcumin and those control treated with DMSO (NS, not statistically significant. Supplementary Figure S6A).

To further examine whether curcumin-upregulated miRNAs inhibit EZH2 expression, we overexpressed each of the miRNAs in A549 cells using transduction with lentiviral particles harboring precursor miRNAs (Supplementary Figure S6B). The measurement of miRNAs in A549 cells demonstrated that a 10- to 40-fold overexpression of the respective miRNA was reached at 72 hours post-transduction (Figure 6B). Next, we measured EZH2 expression in A549 cells with overexpression of a specific miRNA. As expected, both miR-let 7c and miR-101 effectively downregulated EZH2 expression (*P* < 0.05, Figure 6C and 6D), in agreement with previous reports [27, 28]. Although miR-361 was reported to act as a tumor suppressor [29], its (most significant) overexpression in A549 cells did not change the expression level of EZH2 (NS, not statistically significant. Figure 6C and 6D).

### Curcumin regulates NOTCH1 expression through EZH2 and the reciprocal regulation between EZH2 and NOTCH1

**Figure 7:**
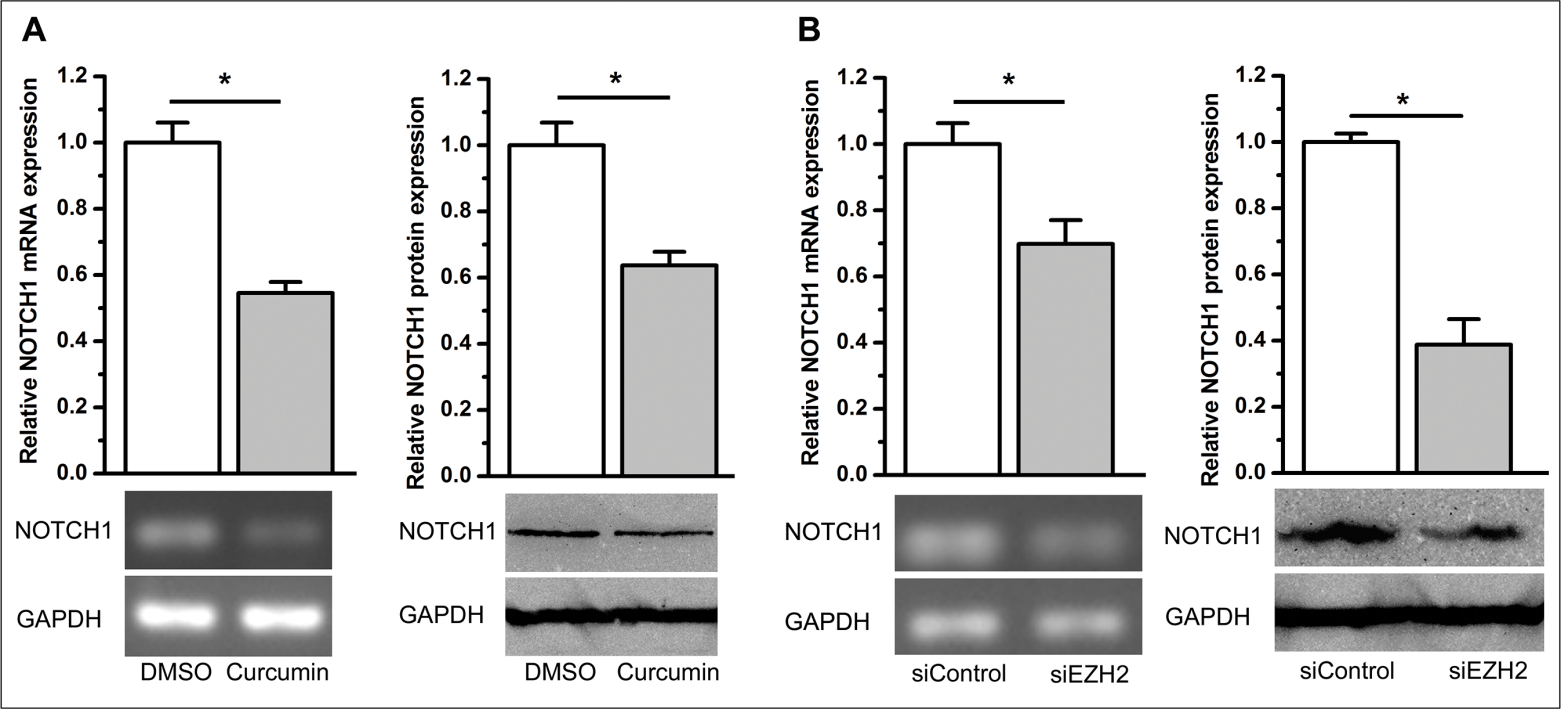
Curcumin and knockdown of EZH2 inhibited NOTCH1 expression (**A**) qPCR (top left) and semi-quantitative PCR (bottom left) demonstrated that curcumin inhibited NOTCH1 mRNA expression. Western blotting (right panel) demonstrated that curcumin inhibited NOTCH1 protein expression. *P < 0.05. The expression level of NOTCH1 mRNA was normalized to ACTB for qPCR analysis. Protein expression levels of NOTCH1 were normalized to GAPDH. Expression levels of NOTCH1 mRNA and protein were normalized to 1 for the control treatment of DMSO. (**B**) qPCR (top left) and semi-quantitative PCR (bottom left) revealed that siEZH2 inhibited NOTCH1 mRNA expression. Western blot analysis (right panel) revealed that siEZH2 inhibits NOTCH1 protein expression. *P < 0.05. The expression level of NOTCH1 mRNA was normalized to ACTB for qPCR test. Protein expression levels of NOTCH1 were normalized to GAPDH. The expression levels of NOTCH1 mRNA and protein were normalized to 1 for the control transfection of siControl. All of the data are plotted as the mean ± SEM from three independent PCR amplifications or western blot assays.

**Figure 8:**
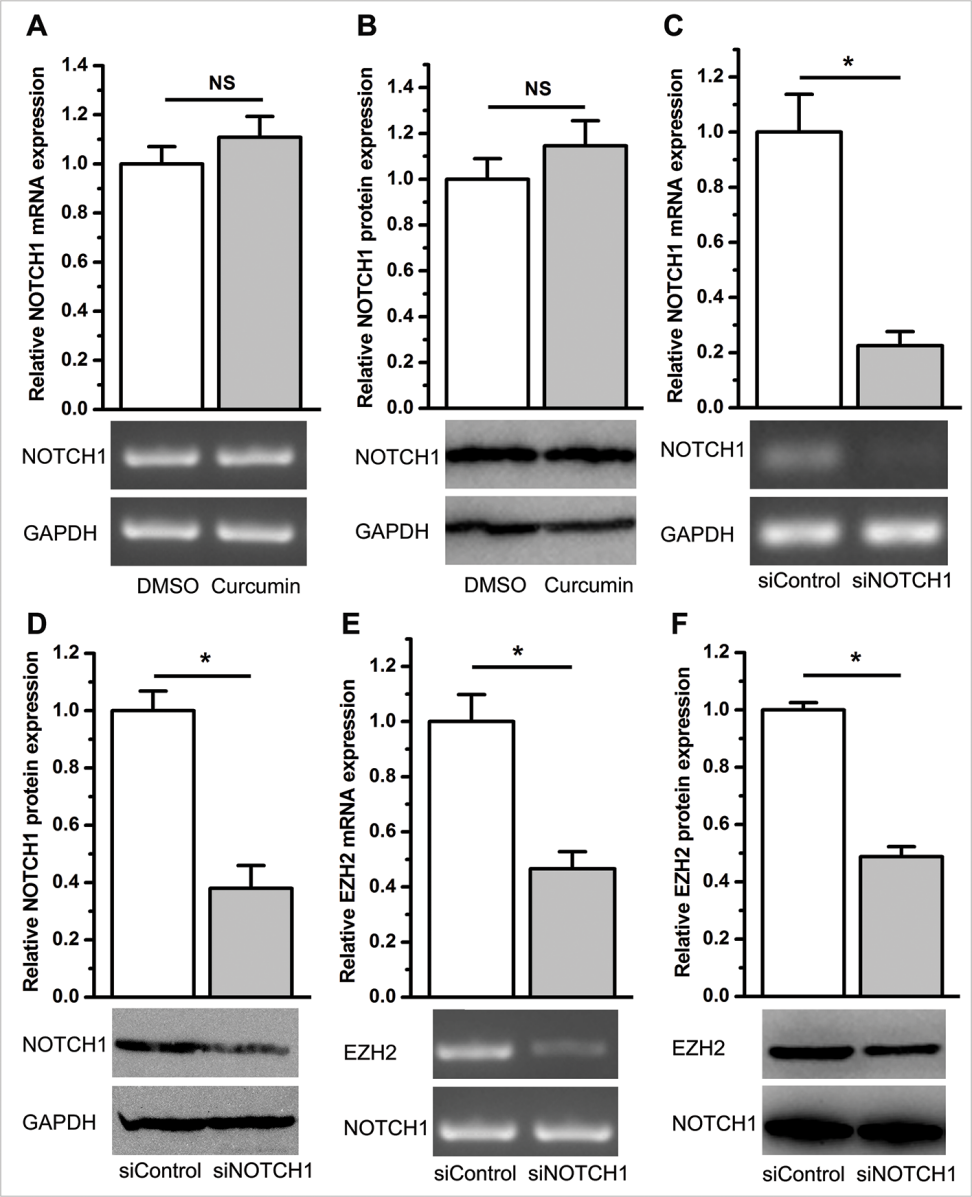
EZH2 abrogated the inhibitory effect of curcumin on NOTCH1 expression, and inhibition of NOTCH1 repressed EZH2 expression (**A**) qPCR (top) and semi-quantitative PCR (bottom) demonstrated that curcumin did not change the expression level of NOTCH1 mRNA in A549 cells stably overexpressing EZH2. NS, not statistically significant. (**B**) Western blot analysis revealed that curcumin did not change the expression level of NOTCH1 protein in A549 cells stably overexpressing EZH2. NS, not statistically significant. (**C**) qPCR (top) and semi-quantitative PCR (bottom) revealed that siNOTCH1 efficiently knocked down NOTCH1 mRNA expression. *P < 0.05. (**D**) Western blot analysis revealed that siNOTCH1 remarkably inhibited NOTCH1 protein expression. *P < 0.05. The expression level of NOTCH1 mRNA was normalized to ACTB for qPCR analysis. Protein expression levels of NOTCH1 were normalized to GAPDH. Expression levels of NOTCH1 mRNA and protein were normalized to 1 for the control treatment of DMSO or the control transfection of siControl. (**E**) qPCR (top) and semi-quantitative PCR (bottom) revealed that siNOTCH1 inhibited EZH2 mRNA expression. (**F**) Western blot analysis revealed that siNOTCH1 inhibits EZH2 protein expression. *P* < 0.05. The expression level of EZH2 mRNA was normalized to ACTB for qPCR analysis. Protein expression levels of EZH2 were normalized to GAPDH. Expression levels of EZH2 mRNA and protein were normalized to 1 for the control transfection of siControl. All of the data are plotted as the mean ± SEM from three independent PCR amplifications or western blot assays.

Based on the oncogenic role of NOTCH1 in cancer development and progression, we hypothesized that NOTCH1 is likely regulated by EZH2. Recently, Gonzalez et al. reported that EZH2 activates NOTCH1 signaling in breast stem cells [30]. This prompted us to further test our hypothesis. Using qPCR and immunoblot analysis, we found that curcumin significantly inhibited NOTCH1 expression (*P* < 0.05, Figure 7A). As expected, we also observed that knockdown of EZH2 significantly inhibited NOTCH1 expression (*P* < 0.05, Figure 7B and Supplementary Figure S7A, S7B). We then overexpressed EZH2 in A549 cells using stable transfection of the *ezh2* plasmid and demonstrated that ectopic overexpression of EZH2 completely abolished curcumin's inhibitory effect on NOTCH1 expression (NS, not statistically significant, Figure 8A and 8B). However, stable overexpression of EZH2 did not change the expression of NOTCH1 (NS, not statistically significant. Supplementary Figure S7C S7D). These results support the idea that curcumin regulates NOTCH1 expression through an inhibition of EZH2.

Given that EZH2 regulates NOTCH1 expression, inhibition of NOTCH1 is expected to cause similar effects as described above in the case of EZH2 inhibition. To understand the functional biology of NOTCH1 in lung cancer cells, we knocked down the expression of NOTCH1 using siRNA (Figure 8C and 8D). As expected, knockdown of NOTCH1 significantly attenuated cell growth, migration and the percentage of cells in the S phase, but increased the percentage of cells in the G_1_ phase, which was consistent with the results from direct inhibition of EZH2 expression (*P* < 0.05, Supplementary Figure S8A–S8F), indicating EZH2′s regulation on NOTCH1.

Finally, we investigated whether NOTCH1 reciprocally regulates EZH2 expression. As determined using qPCR and western blotting, EZH2 was significantly suppressed after RNA interference (RNAi) knockdown of NOTCH1 (*P* < 0.05, Figure 8E and 8F).

## DISCUSSION

Our study is the first to investigate curcumin's regulation of EZH2 and the mechanism by which curcumin inhibits the expression of EZH2 in lung cancer cells. We revealed that both curcumin and RNAi of EZH2 induced cell cycle arrest and significantly lowered the percentage of cells in the S phase. However, knockdown of EZH2 did not exactly duplicate the cell cycle distribution of cells after the treatment with curcumin. In agreement with previous reports [31–33], curcumin significantly arrested cells in the G_2_/M phase, and increased the number of cells in the G_1_ phase, but the latter was not statistically significant. Many studies have shown that EZH2 regulates cell cycle progression through delaying the G_1_/S or G_2_/M cell cycle transition [34–37]. In our study, depletion of EZH2 significantly induced G_1_ arrest, but did not change the percentage of cells in the G_2_/M phase. Moreover, as mentioned above, artificial overexpression of EZH2 increased the tolerance of lung cancer cells against curcumin; however these cells were not able to survive curcumin treatment. Taken together, these results suggest that EZH2 is an important target of curcumin in lung cancer, but it is not the only target.

NOTCH signaling plays a critical role in maintaining the balance of cell proliferation and apoptosis. It has been suggested that NOTCH1 functions as an oncogene or tumor suppressor gene in a cell type-specific manner. Recent studies revealed that an inhibition of NOTCH1 activation or expression is accompanied by cell cycle arrest mainly in the G_1_ phase [38, 39]. While some studies reported that ligand-independent and ligand-dependent activation of NOTCH1 or overexpression of NOTCH1 promotes cell cycle progression [40–42], quite a few studies suggested a tumor suppressive role for NOTCH1 given that the overexpression of the intracellular region of Notch1 (ICN) inhibits cell cycle at G_1_ phase or G_2_ phase [43–45]. It is well established that curcumin suppresses NOTCH signaling [46, 47]. However, it remains unclear whether curcumin regulates NOTCH signaling directly or through the regulation of other molecule(s). Histone deacetylase (HDAC) activity is indispensable for EZH2-mediated gene repression [48], and HDAC inhibitors can induce NOTCH1 expression in some endocrine cancers [49], indicating the relevance of EZH2 and NOTCH1 and their convergence at HDAC. We found that both curcumin and a depletion of EZH2 using siRNA significantly reduced the expression of NOTCH1. In contrast with its controversial roles in the regulation of the cell cycle, it was consistently reported that NOTCH1 promoted cancer cell migration and invasion *in vitro* [41, 50–52]. In the current study, we found that compared with RNAi knockdown of EZH2, knockdown of NOTCH1 mimicked the effects on lung cancer cell proliferation, cell cycle distribution, and cell migration. Furthermore, ectopic overexpression of EZH2 completely abolished the inhibitory effect of curcumin on NOTCH1 expression. Collectively, these observations strongly suggest that EZH2 regulates NOTCH1.

Based on western blot analysis, NOTCH1 protein was only detectable in A549, NCI-H520, and NCI-H1373 cells but not NCI-H2170, although NOTCH1 mRNA was detected using PCR in all four cell lines. This finding suggests that the expression level of NOTCH1 protein in NCI-H2170 cells is too low to be detected using western blotting.

Curcumin significantly suppressed the migration of lung cancer cells through the permeable transwell insert membrane and cell invasion through the Matrigel basement membrane matrix. By comparing the cell proliferation rate, we showed that the viable cell number is not significantly different at 9 hours post cell plating between the curcumin group and the control group. This further confirmed curcumin's inhibition of cell migration. However, the viable cell number in the curcumin group was significantly lower than that of the control group at 72 hours post cell plating. This observation made it difficult to discern whether curcumin inhibits cell invasion *in vitro* as the inhibition of cell proliferation is likely to lower the number of cells invading through the Matrigel basement membrane matrix.

Curcumin inhibited *ezh2* promoter luciferase activity and EZH2 3′ UTR luciferase activity, which had not been previously demonstrated. The miRNA array analysis revealed that curcumin promoted the expression of miR-101 and miR-let 7c, which was confirmed using qPCR. Our recent study found that miR-101 and miR-let 7c inhibited EZH2 3′UTR-luciferase activity [53]. The ectopic overexpression of miR-101 or miR-let 7c inhibited EZH2 expression in lung cancer cells. All of these observations implicate a hitherto unrecognized mechanism by which curcumin regulates the expression of EZH2.

As we previously reported [15], curcumin causes G_1_ arrest of the cell cycle and downregulates the expression of EZH2 through the mitogen-activated protein kinase pathway in breast cancer. In the present study, we found that EZH2 was significantly downregulated by curcumin; however, we were not able to mimic the conditions of breast cancer to reveal the mechanism by which curcumin inhibits EZH2. Instead, similar to other studies, we provided strong evidence that curcumin arrested lung cancer cells in the G_2_/M phase. We also demonstrated that curcumin regulated the expression of EZH2 both transcriptionally and post-transcriptionally. These observations implicate a tumor-type-specific mechanism through which curcumin inhibits the proliferation of cancer cells and the expression of EZH2.

Curcumin acts pro- and anti-apoptotically [54], and depletion of EZH2 induces cell apoptosis [55, 56]. Moreover, extensive studies indicate that NOTCH signaling is either anti-apoptotic [57, 58] or pro-apoptotic [59]. However, neither curcumin nor RNAi against EZH2 or NOTCH1 could induce cell apoptosis in the lung cancer cells that we investigated.

Although RNAi inhibition of EZH2 induced cell cycle arrest and suppressed the expression of NOTCH1, overexpression of EZH2 in A549 cells did not induce changes to the cell cycle or to NOTCH1 expression. These observations suggest that the endogenous expression level of EZH2 is sufficient for EZH2′s physiological function and that the artificial overexpression of EZH2 is redundant.

RNAi knockdown of EZH2 significantly inhibited the expression of NOTCH1 and vice versa; knocking down NOTCH1 significantly decreased the expression of EZH2. These findings indicate that EZH2 and NOTCH1 are reciprocally regulated.

To the best of our knowledge, the current study is the first to report that curcumin regulates the expression of EZH2 in lung cancer. We have provided extensive evidence of this regulation. For example, curcumin inhibits the transcription of the *ezh2* gene; curcumin degrades EZH2 mRNA through elevated levels of miR-101 and miR-let 7c; and the decreased level of EZH2 resulted in the downregulation of NOTCH1 in lung cancer. In conclusion, curcumin inhibits lung cancer growth through, at least in part, the inhibition of EZH2 and its reciprocally regulated molecule NOTCH1, suggesting that the molecules that are involved in the EZH2/NOTCH signaling pathway are potential therapeutic targets for lung cancer. Curcumin upregulated the expression of miR-361, but we failed to correlate this phenomenon with the regulation of EZH2 by curcumin. Future studies are warranted to explore the role of miR-361 in the development of lung cancer and the involvement of miR-361 in the mechanism through which curcumin suppresses lung cancer.

## MATERIALS AND METHODS

### Cell culture and lentiviral transduction

The human lung cancer cell lines A549, NCI-H520, NCI-H1373, and NCI-H2170 were obtained from the American Type Culture Collection (ATCC, Manassas, VA), where the cell lines were authenticated using STR profiling before distribution. Cells were maintained in RPMI 1640 supplemented with 10% fetal bovine serum (FBS, Gibco/Life Technologies, Grand Island, NY) in a humidified atmosphere containing 5% CO_2_ at 37°C.

A549 cells that grew in 25-cm^2^ flasks were infected with lentiviral particles expressing miR-let 7c (LV-miR-let 7c), miR-101 (LV-miR-101), miR-361 (LV-miR-361), or the control virus (LV-Control) (Invitrogen, Shanghai, China) at a multiplicity of infection of 15. At 16 hours after the transduction, the medium was replaced and incubated at 37°C in a humidified atmosphere of 5% CO_2_ for a further 72 hours followed by subsequent experiments.

### Plasmid construction

We amplified the EZH2 coding sequence from pCMV6-EZH2 (Origene, Rockville, MD) using primer 1 (Supplementary Table S1) and cloned it into pcDNA3.1(+) (Invitrogen, Carlsbad, CA) between the NheI and KpnI sites to construct the vector expressing EZH2 (pcDNA3-EZH2Flag). *ezh2* promoter (−1772 to +112 relative to the start of the first exon) amplified with primer 2 (Supplementary Table S1) from genomic DNA of A549 cells or the randomly scrambled *ezh2* promoter that was synthesized was inserted into the NheI and HindIII sites of pGL3-Basic (Promega, Madison, WI) to generate the *ezh2* promoter luciferase reporter vector pGL3-EZH2P and the negative control vector pGL3-EZH2Pscram, respectively. To construct pMIR-EZH2 UTR, 263 bp of the EZH2 3′ UTR sequence was amplified and cloned into the luciferase reporter vector pMIR-REPORT^™^ Luciferase (Ambion/Life Technologies, Grand Island, NY) between the SpeI and HindIII sites as previously reported [18]. The scrambled EZH2 3′ UTR was cloned into the multiple cloning site of pMIR-REPORT^™^ Luciferase to generate the control construct pMIR-EZH2 UTRscram.

### Cell transfection

A549 cells were transfected with pcDNA3-EZH2Flag using Lipofectamine^™^ 2000 (Invitrogen, Carlsbad, CA) according to the manufacturer's instructions. Cells were passaged at 1:10 dilution 24 hours after transfection. After another 24 hours, 0.5 mg/ml of G418 (CALBIOCHEM, Billerica, MA) was added to the culture medium to select cell lines that stably expressed EZH2.

A549 cells were transfected with siEZH2 or siRNA against NOTCH1 (siNOTCH1) (Invitrogen, Carlsbad, CA) (Supplementary Table S1) using Lipofectamine^™^ 2000 according to the manufacturer's instructions, and the subsequent experiments were performed 72 hours later.

### Cell proliferation assays

Lung cancer cells were plated in 25-cm^2^ flasks rather than in cell culture plates or cell culture dishes to avoid the misdistribution of cells on the bottom of the culture vessels. Each flask was filled with 5 ml of cell suspension at a concentration of 1 × 10^5^ cells/ml. For the proliferation assay with curcumin, a final concentration of 6 μM of curcumin (Sigma-Aldrich, Saint Louis, MO) or an equivalent volume of DMSO (Amresco, Solon, OH) was added to the cell culture media 6 hours post cell plating. After incubation for 72 hours, cells were observed under a microscope and photographed, followed by digestion with 0.25% trypsin and 0.02% ethylenediaminetetraacetic acid (EDTA) (Genom, Hangzhou, China), resuspension, 0.2% trypan blue (Dingguo, Beijing, China) staining, and enumeration of viable cells with a Countstar automated cell counter (Ruiyu Biotechnology, Shanghai, China).

### Cell cycle analysis and measurement of apoptosis

A549 cells that were transfected with siRNAs, or incubated in media containing 6 μM of curcumin or an equivalent volume of DMSO for 72 hours were trypsinized and fixed by 70% ethanol followed by staining using a Coulter DNAPrep Reagents Kit (Beckman Coulter, Fullerton, CA). The cellular DNA content from each sample was determined using a FACScan apparatus (Becton Coulter, Fullerton, CA). All of the experiments were performed in triplicate.

For the characterization of the cell cycle using anti-bromodeoxyuridine (BrdU) and propidium iodide (PI) staining, suspend A549 cells in 60-mm dishes at a concentration of 10^6^ cells/dish. Twenty-four hours later, transfected cells with siRNAs or incubate cells in medium containing curcumin. Another 24 hours later, add BrdU to the culture medium to achieve a final concentration of 50 μM followed by incubation for 1 hour in the CO_2_ incubator at 37°C. Resuspend cells in phosphate-buffered saline (PBS), and add chilled ethanol to achieve a final concentration of 70% (v/v). Store cells at 4°C overnight. Wash cells with PBS and add 1 ml of 2 M Hydrochloric acid followed by incubation at room temperature (RT) for 1 hour. Wash cells with PBS and add 2 μl of anti-BrdU (Roche, Mannheim, Germany). Incubate cells at RT overnight. Wash cells with blocking buffer and add 2 μl of goat anti-mouse IgG FITC (MultiSciences, Hangzhou, China). Incubate cells in the dark at RT for 3 hours. Wash cells with PBS and add 500 μl of DNA PREP Stain (Beckman Coulter, Brea, CA). Incubate cells in the dark at RT for 30 minutes followed by analyzing on a FACScan apparatus.

Apoptotic cells were evaluated using an Annexin V-EGFP/PI Kit (Keygentec, Nanjing, China) based on the manufacturer's instructions. Briefly, A549 cells incubated in media with 6 μM of curcumin or an equivalent volume of DMSO for 72 hours were trypsinized with 0.25% trypsin in the absence of EDTA. The cells were washed with PBS twice and resuspended in 500 μl of binding buffer at a concentration of 0.2–1.0 × 10^6^ cells/ml. Five microliters of annexin V-EGFP and 5 μl of PI (Sigma-Aldrich, Saint Louis, MO) were added to the suspension followed by 10 to 20-minutes of incubation in the dark. The cells were then analyzed using the FACS can apparatus.

### Cell migration and invasion assays

A transwell permeable supports system (Corning Incorporated, Corning, NY) was used to perform cell migration assays. A549 cells that were incubated in media with 6 μM of curcumin or an equivalent volume of DMSO for 72 hours were resuspended in RPMI 1640 supplemented with 1% FBS to a final concentration of 2.5 × 10^5^ cells/ml. Each lower chamber of the transwell plate was filled with 600 μl of complete culture media (RPMI1640 supplemented with 10% FBS). Each upper chamber was loaded with 100 μl of A549 cell suspension before being transferred back to the lower chamber. The chambers were then incubated at 37°C for 9, 12 and 15 hours, followed by removal of the cells on the upper surface of the upper chamber membrane using cotton swabs. Finally, the membrane was fixed and stained with 1% toluidine blue (Dingguo, Beijing, China) before cell counting under a microscope. A total of ten fields were counted for each chamber.

Cell invasion assays were performed using a BD BioCoat^™^ Matrigel^™^ Invasion Chamber (BD Biosciences, Bedford, MA) according to the manufacturer's instructions. Briefly, the A549 cells described above were resuspended in RPMI 1640 supplemented with 1% FBS to a final concentration of 5 × 10^4^ cells/ml. Each lower chamber of the plate and insert was filled with 750 μl of complete culture media and 500 μl of A549 cell suspension, respectively. After incubation at 37°C for 48, 72 and 96 hours, the Matrigel inserts were then processed in the procedure for the cell migration assays that is noted in the previous paragraph.

A549 cells were also plated in a 24-well plate at the concentration of 2.5 × 10^4^ cells/well in 1 ml of medium supplemented with 1% or 10% FBS followed by incubation for 9, 12, 15, 48, 72, and 96 hours. Next, cells were harvested by digestion with 0.25% trypsin and 0.02% EDTA, stained with 0.2% trypan blue, and counted for viable cells with a Countstar automated cell counter.

### Luciferase reporter assays

A549 cells were cultured at 2 × 10^4^ cells/well in the 96-well culture plate and co-transfected with 0.2 μg of the luciferase reporter construct (pGL3-EZH2P, pGL3-EZH2Pscram, pMIR-EZH2 UTR, or pMIR-EZH2 UTRscram) and the internal control vector pRL-TK (Promega, Madison, WI) in a ratio of 10:1 for reporter construct:control vector using Lipofectamine^™^ 2000 according to the manufacturer's transfection procedure. Six hours post-transfection, the transfection medium was removed and replenished with medium containing 6 μM of curcumin or an equivalent volume of DMSO. Forty-eight hours post transfection, luciferase activity was measured using the Dual-Luciferase^®^ Reporter Assay System (Promega). Firefly luciferase activity was normalized to that of Renilla luciferase.

### miRNA expression profiling

For miRNA expression profiling, total RNA was isolated using an RNeasy Mini Kit (Qiagen, Hilden, Germany) after the cells were cultured for 72 hours in medium with curcumin or DMSO. Global miRNA profiling was performed using the TaqMan Low Density Array (TLDA) Human miRNA Panel version 3.0 (Invitrogen, Shanghai, China). TaqMan real-time PCR was carried out as described by the manufacturer (Invitrogen, Shanghai, China) to confirm the miRNA changes revealed in miRNA array analysis. The relative fold change in miRNA expression was calculated using the 2^−ΔΔCT^ method, where the average of ^Δ^CT values for the amplicon of interest was normalized to that of the U6 promoter, and compared with the control specimens.

### Semi-quantitative PCR and quantitative real-time PCR (qPCR)

Lung cancer cells were treated with curcumin, transfected with 30 nM siEZH2 or siNOTCH1, and at the indicated time points, the cells were harvested for the isolation of total RNA using the RNeasy Mini Kit. Reverse transcription was performed using the PrimeScript II 1st Strand cDNA Synthesis Kit (TaKaRa, Dalian, China) according to the manufacturer's recommendations. The primer sequences used in this study are listed in Supplementary Table S1. Semi-quantitative PCR for EZH2, NOTCH1, and glyceraldehyde-3-phosphate dehydrogenase (GAPDH) were cycled as follows: 94°C, 30 s → 55°C, 30 s → 72°C, 30 s for 22 to 36 cycles followed by incubation at 72°C for 5 min.

qPCR were performed using the ABI PRISM 7900 Sequence Detection System (Applied Biosystems, Foster City, CA). The relative fold changes in mRNA expression were calculated using the 2^−ΔΔCT^ method, where the average of ^Δ^CT values for the amplicon of interest was normalized to that of actin beta (ACTB), and compared with the control specimens.

### Western blot

Lung cancer cell lysates were prepared using RIPA buffer (BestBio, Shanghai, China) and equalized for protein concentrations with a BCA Kit (Pierce, Rockford, IL) according to the manufacturers' recommendations. An equal amount of whole cell lysates were resolved using SDS-polyacrylamide gel electrophoresis and transferred onto a polyvinylidene difluoride membrane (Bio-Rad, Hercules, CA) followed by incubation with primary mouse monoclonal antibodies against human EZH2 (BD Biosciences), flag (Multisciences, Hangzhou, China), rabbit polyclonal antibody against human GAPDH (Santa Cruz Biotechnology, Santa Cruz, CA), rabbit monoclonal antibody against human NOTCH1 (Abcam, Shanghai, China) and goat anti-mouse or anti-rabbit IgG-HRP (Santa Cruz Biotechnology). The immunoreactive proteins were detected with SuperSignal^®^ West Pico Chemiluminescent Substrate (Thermo, Rockford, IL) according to the manufacturer's instructions.

### Statistical analysis

The cell proliferation data of A549 cells with EZH2 overexpressed were analyzed using a 2-tailed Student's *t*-test. The remaining data were analyzed using Mann-Whitney-Wilcoxon test. The differences were considered to be statistically significant at a *P* value less than 0.05.

## SUPPLEMENTARY MATERIALS FIGURES AND TABLE



## References

[1] Jemal A, Bray F, Center MM, Ferlay J, Ward E, Forman D (2011). Global cancer statistics. CA Cancer J Clin.

[2] Ettinger DS (2012). Ten years of progress in non-small cell lung cancer. J Natl Compr Canc Netw.

[3] Jett JR, Carr LL (2013). Targeted therapy for non-small cell lung cancer. Am J Respir Crit Care Med.

[4] Howlader N, Noone AM, Krapcho M, Garshell J, Miller D, Altekruse SF, Kosary CL, Yu M, Ruhl J, Tatalovich Z, Mariotto A, Lewis DR, Chen HS SEER Cancer Statistics Review, 1975–2011.

[5] Strimpakos AS, Sharma RA (2008). Curcumin: preventive and therapeutic properties in laboratory studies and clinical trials. Antioxid Redox Signal.

[6] Hong RL, Spohn WH, Hung MC (1999). Curcumin inhibits tyrosine kinase activity of p185neu and also depletes p185neu. Clinical Cancer Research.

[7] Chen A, Xu J, Johnson AC (2005). Curcumin inhibits human colon cancer cell growth by suppressing gene expression of epidermal growth factor receptor through reducing the activity of the transcription factor Egr-1. Oncogene.

[8] Hong J, Bose M, Ju J, Ryu JH, Chen X, Sang S, Lee MJ, Yang CS (2004). Modulation of arachidonic acid metabolism by curcumin and related beta-diketone derivatives: effects on cytosolic phospholipase A2, cyclooxygenases and 5-lipoxygenase. Carcinogenesis.

[9] Banning A, Deubel S, Kluth D, Zhou Z, Brigelius-Flohe R (2005). The GI-GPx gene is a target for Nrf2. Molecular and Cellular Biology.

[10] Aggarwal BB (2003). Signalling pathways of the TNF superfamily: a double-edged sword. Nature Reviews Immunology.

[11] Shishodia S, Amin HM, Lai R, Aggarwal BB (2005). Curcumin (diferuloylmethane) inhibits constitutive NF-kappa B activation, induces G1/S arrest, suppresses proliferation, and induces apoptosis in mantle cell lymphoma. Biochemical Pharmacology.

[12] Philip S, Kundu GC (2003). Osteopontin induces nuclear factor kappa B-mediated promatrix metalloproteinase-2 activation through I kappa B alpha/IKK signaling pathways, and curcumin (diferulolylmethane) down-regulates these pathways. Journal of Biological Chemistry.

[13] Han SS, Chung ST, Robertson DA, Ranjan D, Bondada S (1999). Curcumin causes the growth arrest and apoptosis of B cell lymphoma by downregulation of egr-1, c-myc, bcl-XL, NF-kappa B, and p53. Clin Immunol.

[14] Liontas A, Yeger H (2004). Curcumin and resveratrol induce apoptosis and nuclear translocation and activation of p53 in human neuroblastoma. Anticancer Research.

[15] Hua WF, Fu YS, Liao YJ, Xia WJ, Chen YC, Zeng YX, Kung HF, Xie D (2010). Curcumin induces down-regulation of EZH2 expression through the MAPK pathway in MDA-MB-435 human breast cancer cells. Eur J Pharmacol.

[16] Cao Q, Mani RS, Ateeq B, Dhanasekaran SM, Asangani IA, Prensner JR, Kim JH, Brenner JC, Jing X, Cao X, Wang R, Li Y, Dahiya A (2011). Coordinated regulation of polycomb group complexes through microRNAs in cancer. Cancer Cell.

[17] Tong ZT, Cai MY, Wang XG, Kong LL, Mai SJ, Liu YH, Zhang HB, Liao YJ, Zheng F, Zhu W, Liu TH, Bian XW, Guan XY (2012). EZH2 supports nasopharyngeal carcinoma cell aggressiveness by forming a co-repressor complex with HDAC1/HDAC2 and Snail to inhibit E-cadherin. Oncogene.

[18] Zheng F, Liao YJ, Cai MY, Liu YH, Liu TH, Chen SP, Bian XW, Guan XY, Lin MC, Zeng YX (2012). The putative tumour suppressor microRNA-124 modulates hepatocellular carcinoma cell aggressiveness by repressing ROCK2 and EZH2. Gut.

[19] Cai MY, Tong ZT, Zheng F, Liao YJ, Wang Y, Rao HL, Chen YC, Wu QL, Liu YH, Guan XY, Lin MC, Zeng YX, Kung HF (2011). EZH2 protein: a promising immunomarker for the detection of hepatocellular carcinomas in liver needle biopsies. Gut.

[20] Bauge C, Bazille C, Girard N, Lhuissier E, Boumediene K (2014). Histone methylases as novel drug targets: developing inhibitors of EZH2. Future Med Chem.

[21] Fillmore CM, Xu C, Desai PT, Berry JM, Rowbotham SP, Lin YJ, Zhang H, Marquez VE, Hammerman PS, Wong KK, Kim CF (2015). EZH2 inhibition sensitizes BRG1 and EGFR mutant lung tumours to TopoII inhibitors. Nature.

[22] Wang X, Wang Q, Ives KL, Evers BM (2006). Curcumin inhibits neurotensin-mediated interleukin-8 production and migration of HCT116 human colon cancer cells. Clin Cancer Res.

[23] Yodkeeree S, Chaiwangyen W, Garbisa S, Limtrakul P (2009). Curcumin, demethoxycurcumin and bisdemethoxycurcumin differentially inhibit cancer cell invasion through the down-regulation of MMPs and uPA. J Nutr Biochem.

[24] Zhou DH, Wang X, Yang M, Shi X, Huang W, Feng Q (2011). Combination of Low Concentration of (-)-Epigallocatechin Gallate (EGCG) and Curcumin Strongly Suppresses the Growth of Non-Small Cell Lung Cancer *in vitro* and *in vivo* through Causing Cell Cycle Arrest. Int J Mol Sci.

[25] Lee SJ, Langhans SA (2012). Anaphase-promoting complex/cyclosome protein Cdc27 is a target for curcumin-induced cell cycle arrest and apoptosis. BMC cancer.

[26] Crea F, Fornaro L, Bocci G, Sun L, Farrar WL, Falcone A, Danesi R (2012). EZH2 inhibition: targeting the crossroad of tumor invasion and angiogenesis. Cancer Metastasis Rev.

[27] Varambally S, Cao Q, Mani R-S, Shankar S, Wang X, Ateeq B, Laxman B, Cao X, Jing X, Ramnarayanan K (2008). Genomic loss of microRNA-101 leads to overexpression of histone methyltransferase EZH2 in cancer. Science.

[28] Bao B, Ali S, Banerjee S, Wang Z, Logna F, Azmi AS, Kong D, Ahmad A, Li Y, Padhye S, Sarkar FH (2012). Curcumin analogue CDF inhibits pancreatic tumor growth by switching on suppressor microRNAs and attenuating EZH2 expression. Cancer Res.

[29] Liu D, Tao T, Xu B, Chen S, Liu C, Zhang L, Lu K, Huang Y, Jiang L, Zhang X, Huang X, Zhang L, Han C (2014). MiR-361-5p acts as a tumor suppressor in prostate cancer by targeting signal transducer and activator of transcription-6(STAT6). Biochem Biophys Res Commun.

[30] Gonzalez ME, Moore HM, Li X, Toy KA, Huang W, Sabel MS, Kidwell KM, Kleer CG (2013). EZH2 expands breast stem cells through activation of NOTCH1 signaling. Proc Natl Acad Sci U S A.

[31] Yan C, Jamaluddin MS, Aggarwal B, Myers J, Boyd DD (2005). Gene expression profiling identifies activating transcription factor 3 as a novel contributor to the proapoptotic effect of curcumin. Mol Cancer Ther.

[32] Chen A, Xu J, Johnson AC (2006). Curcumin inhibits human colon cancer cell growth by suppressing gene expression of epidermal growth factor receptor through reducing the activity of the transcription factor Egr-1. Oncogene.

[33] Su CC, Lin JG, Li TM, Chung JG, Yang JS, Ip SW, Lin WC, Chen GW (2006). Curcumin-induced apoptosis of human colon cancer colo 205 cells through the production of ROS, Ca2+ and the activation of caspase-3. Anticancer Res.

[34] Bracken AP, Pasini D, Capra M, Prosperini E, Colli E, Helin K (2003). EZH2 is downstream of the pRB-E2F pathway, essential for proliferation and amplified in cancer. Embo J.

[35] Otte AP, Kwaks TH (2003). Gene repression by Polycomb group protein complexes: a distinct complex for every occasion?. Curr Opin Genet Dev.

[36] Kleer CG, Cao Q, Varambally S, Shen R, Ota I, Tomlins SA, Ghosh D, Sewalt RG, Otte AP, Hayes DF, Sabel MS, Livant D, Weiss SJ (2003). EZH2 is a marker of aggressive breast cancer and promotes neoplastic transformation of breast epithelial cells. Proc Natl Acad Sci U S A.

[37] Gonzalez ME, Li X, Toy K, DuPrie M, Ventura AC, Banerjee M, Ljungman M, Merajver SD, Kleer CG (2009). Downregulation of EZH2 decreases growth of estrogen receptor-negative invasive breast carcinoma and requires BRCA1. Oncogene.

[38] Carlesso N, Aster JC, Sklar J, Scadden DT (1999). Notch1-induced delay of human hematopoietic progenitor cell differentiation is associated with altered cell cycle kinetics. Blood.

[39] Xu P, Qiu M, Zhang Z, Kang C, Jiang R, Jia Z, Wang G, Jiang H, Pu P (2010). The oncogenic roles of Notch1 in astrocytic gliomas *in vitro* and *in vivo*. J Neurooncol.

[40] Sarmento LM, Huang H, Limon A, Gordon W, Fernandes J, Tavares MJ, Miele L, Cardoso AA, Classon M, Carlesso N (2005). Notch1 modulates timing of G1-S progression by inducing SKP2 transcription and p27 Kip1 degradation. J Exp Med.

[41] Ai Q, Ma X, Huang Q, Liu S, Shi T, Zhang C, Zhu M, Zhang Y, Wang B, Ni D, Li H, Zheng T, Zhang X (2012). High-level expression of Notch1 increased the risk of metastasis in T1 stage clear cell renal cell carcinoma. PLoS One.

[42] Xu L, Zhu Y, Xu J, Wu K, Li J, Xu W, Liu H, Wang S, Yin H, Chen L, Wang G, Lin Z (2012). Notch1 activation promotes renal cell carcinoma growth via PI3K/Akt signaling. Cancer Sci.

[43] Morimura T, Goitsuka R, Zhang Y, Saito I, Reth M, Kitamura D (2000). Cell cycle arrest and apoptosis induced by Notch1 in B cells. J Biol Chem.

[44] Wang C, Qi R, Li N, Wang Z, An H, Zhang Q, Yu Y, Cao X (2009). Notch1 signaling sensitizes tumor necrosis factor-related apoptosis-inducing ligand-induced apoptosis in human hepatocellular carcinoma cells by inhibiting Akt/Hdm2-mediated p53 degradation and up-regulating p53-dependent DR5 expression. J Biol Chem.

[45] Qi R, An H, Yu Y, Zhang M, Liu S, Xu H, Guo Z, Cheng T, Cao X (2003). Notch1 signaling inhibits growth of human hepatocellular carcinoma through induction of cell cycle arrest and apoptosis. Cancer Res.

[46] Wang Z, Zhang Y, Banerjee S, Li Y, Sarkar FH (2006). Notch-1 down-regulation by curcumin is associated with the inhibition of cell growth and the induction of apoptosis in pancreatic cancer cells. Cancer.

[47] Subramaniam D, Ponnurangam S, Ramamoorthy P, Standing D, Battafarano RJ, Anant S, Sharma P (2012). Curcumin induces cell death in esophageal cancer cells through modulating Notch signaling. PLoS One.

[48] Varambally S, Dhanasekaran SM, Zhou M, Barrette TR, Kumar-Sinha C, Sanda MG, Ghosh D, Pienta KJ, Sewalt RG, Otte AP, Rubin MA, Chinnaiyan AM (2002). The polycomb group protein EZH2 is involved in progression of prostate cancer. Nature.

[49] Xiao X, Ning L, Chen H (2009). Notch1 mediates growth suppression of papillary and follicular thyroid cancer cells by histone deacetylase inhibitors. Mol Cancer Ther.

[50] Yeh TS, Wu CW, Hsu KW, Liao WJ, Yang MC, Li AF, Wang AM, Kuo ML, Chi CW (2009). The activated Notch1 signal pathway is associated with gastric cancer progression through cyclooxygenase-2. Cancer Res.

[51] Hsu KW, Hsieh RH, Huang KH, Fen-Yau Li A, Chi CW, Wang TY, Tseng MJ, Wu KJ, Yeh TS (2012). Activation of the Notch1/STAT3/Twist signaling axis promotes gastric cancer progression. Carcinogenesis.

[52] Su BH, Qu J, Song M, Huang XY, Hu XM, Xie J, Zhao Y, Ding LC, She L, Chen J, Lin LS, Lin X, Zheng DL (2014). NOTCH1 signaling contributes to cell growth, anti-apoptosis and metastasis in salivary adenoid cystic carcinoma. Oncotarget.

[53] Wu GQ, Wang X, Zhou HY, Chai KQ, Xue Q, Zheng AH, Zhu XM, Xiao JY, Ying XH, Wang FW, Rui T, Xu LY, Zhang YK (2015). Evidence for transcriptional interference in a dual-luciferase reporter system. Sci Rep.

[54] Pari L, Tewas D, Eckel J (2008). Role of curcumin in health and disease. Arch Physiol Biochem.

[55] Wu ZL, Zheng SS, Li ZM, Qiao YY, Aau MY, Yu Q (2010). Polycomb protein EZH2 regulates E2F1-dependent apoptosis through epigenetically modulating Bim expression. Cell Death Differ.

[56] Zhang Q, Padi SK, Tindall DJ, Guo B (2014). Polycomb protein EZH2 suppresses apoptosis by silencing the proapoptotic miR-31. Cell Death Dis.

[57] Liu WH, Hsiao HW, Tsou WI, Lai MZ (2007). Notch inhibits apoptosis by direct interference with XIAP ubiquitination and degradation. Embo J.

[58] Meurette O, Stylianou S, Rock R, Collu GM, Gilmore AP, Brennan K (2009). Notch activation induces Akt signaling via an autocrine loop to prevent apoptosis in breast epithelial cells. Cancer Res.

[59] Zweidler-McKay PA, He Y, Xu L, Rodriguez CG, Karnell FG, Carpenter AC, Aster JC, Allman D, Pear WS (2005). Notch signaling is a potent inducer of growth arrest and apoptosis in a wide range of B-cell malignancies. Blood.

