# Supplementary material for: Anti-cancer effects of curcumin on lung cancer through the inhibition of EZH2 and NOTCH1
Source: Oncotarget. 2016 Apr 1;7(18):26535–50. doi: 10.18632/oncotarget.8532 (PMC5041997; doi:10.18632/oncotarget.8532)
Supplement: Supplementary file 1 [file oncotarget-07-26535-s001.pdf]

Anti-cancer effects of curcumin on lung cancer through the inhibition of EZH2 and NOTCH1

Supplementary Materials

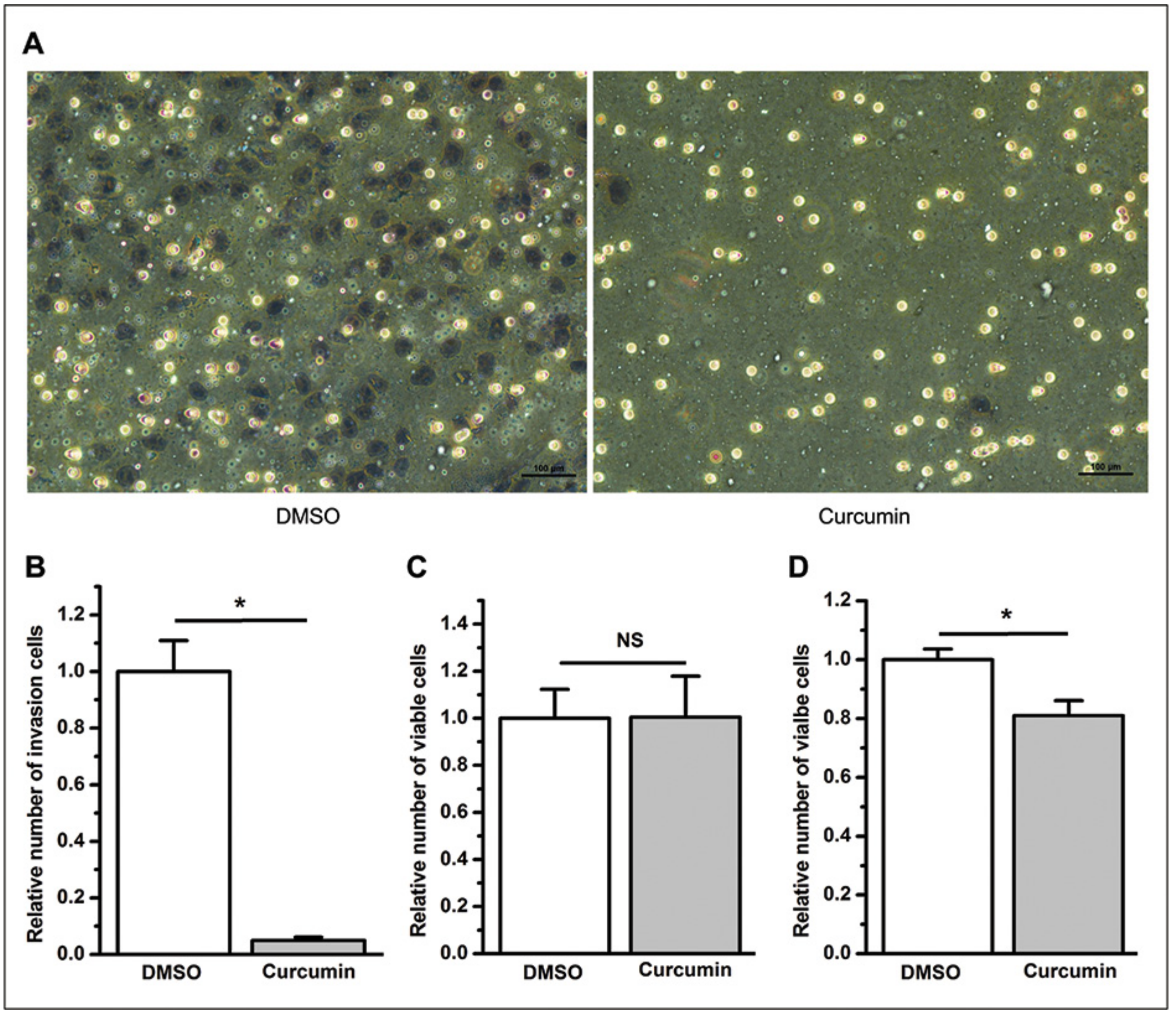

Supplementary Figure S1: The impact of curcumin on lung cancer cell invasion and proliferation capacity. (**A**) Compared to the control treatment of DMSO (left), curcumin inhibited the invasion of A549 cells through the Matrigel basement membrane matrix of cell culture inserts (right). The cell invasion assay was repeated three times with similar results, and triplicate inserts were included in each experiment. (**B**) The relative number of cells that invaded through the Matrigel basement membrane matrix. $*P < 0.05$. The number of invasion cells in the curcumin group was normalized to 1 for the DMSO group. The cell invasion assay was repeated three times with similar results, and triplicate inserts were included in each experiment. The data are plotted as the mean ± SEM. (**C**) When incubated in medium with 1% FBS, the cell proliferation rate of A549 cells treated with DMSO and that treated with curcumin was similar at 9 hours after cell plating in a 24-well plate. NS, not statistically significant. (**D**) The cell proliferation rate of A549 cells treated with DMSO was higher than that treated with curcumin at 72 hours after cell plating in a 24-well plate. $*P < 0.05$. The cell proliferation assay was performed three times with similar results, and triplicate wells were included in each experiment. The data are plotted as the mean ± SEM.

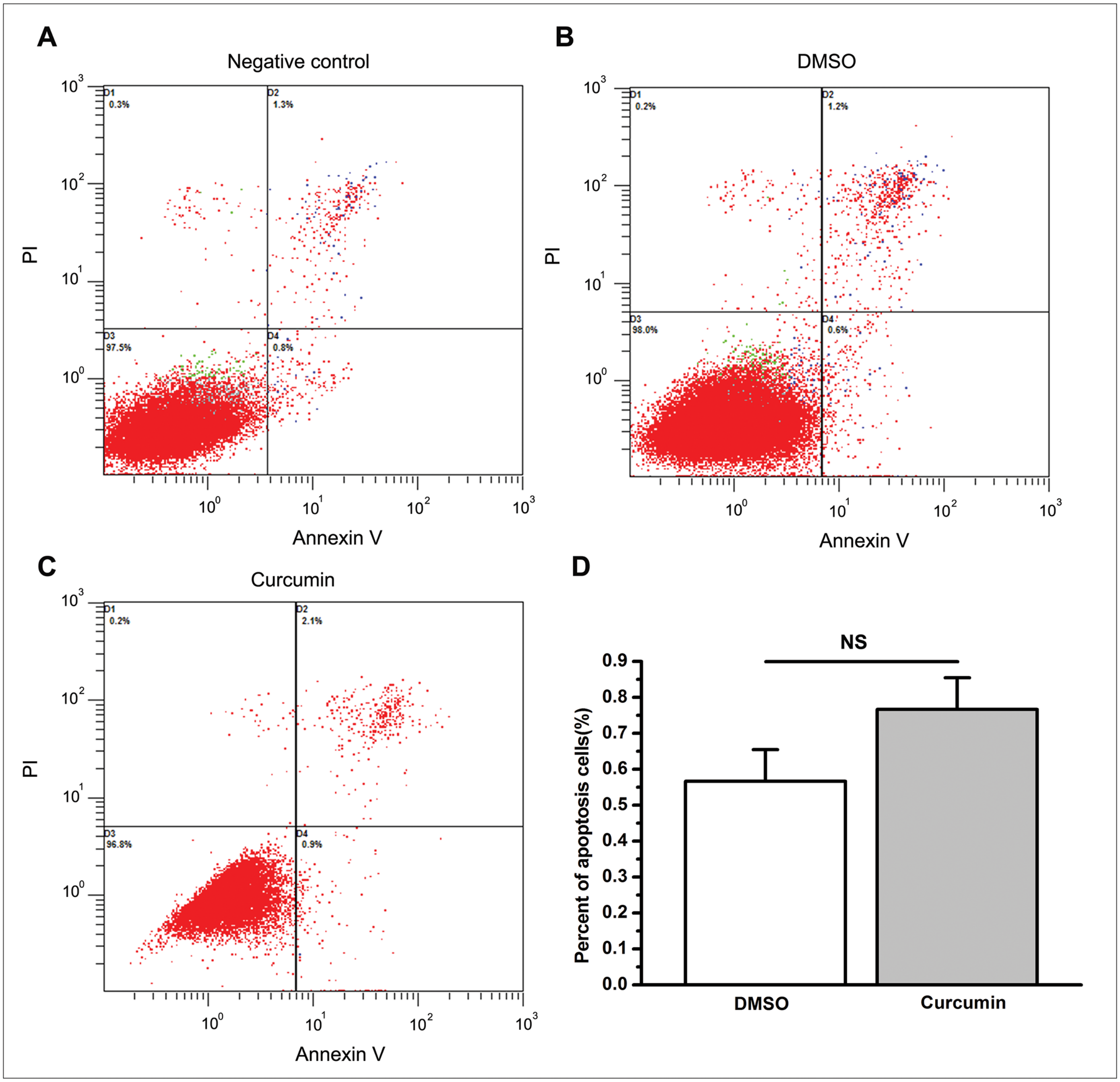

Supplementary Figure S2: Curcumin did not induce cell apoptosis. (**A**) Representative Annexin V-PI staining of A549 cells without treatment with curcumin or DMSO. (**B**) Representative Annexin V-PI staining of A549 cells treated with DMSO for 72 hours. (**C**) Representative Annexin V-PI staining of A549 cells treated with curcumin for 72 hours. (**D**) The percentages of apoptotic cells between the DMSO and curcumin group were not significantly different. NS, not statistically significant. The cell apoptosis assay was performed three times with similar results, and triplicate vessels were included in each experiment. The data are plotted as the mean ± SEM.

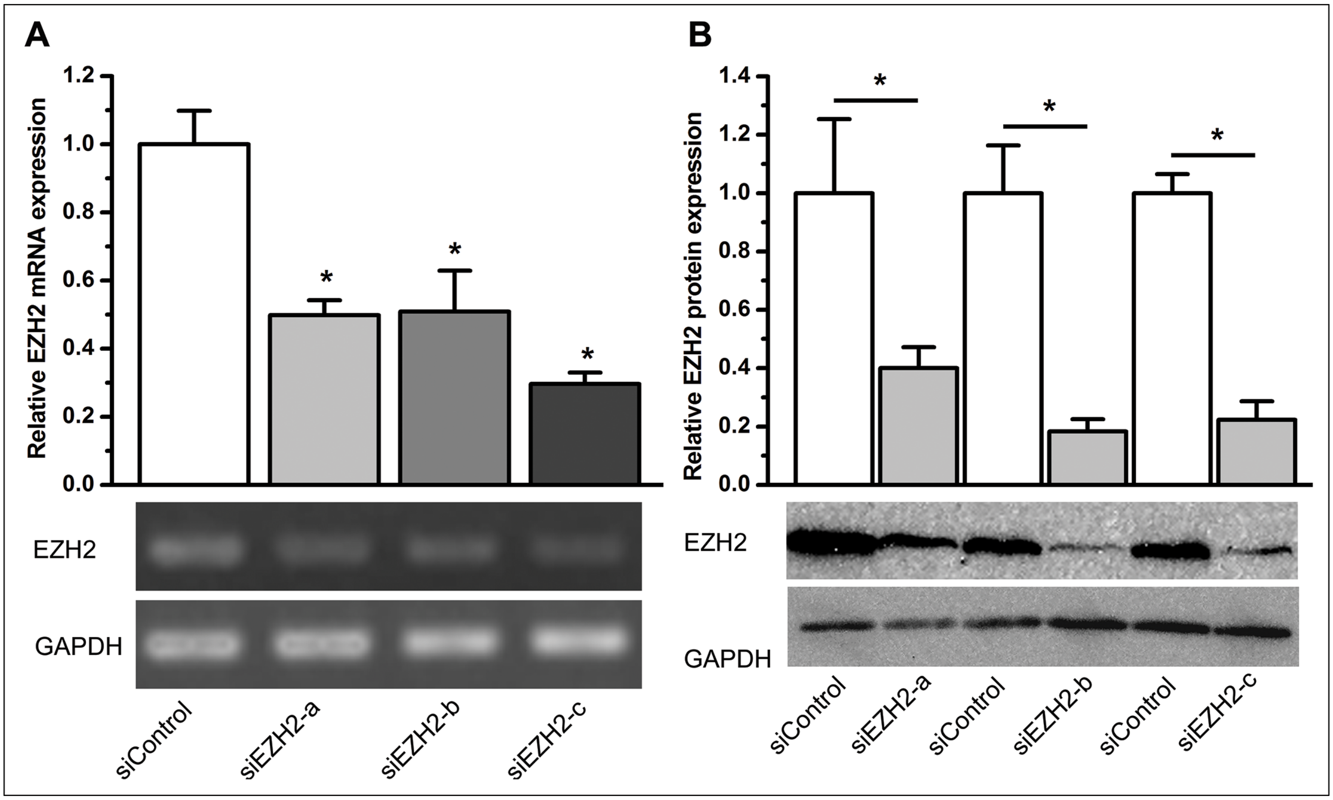

Supplementary Figure S3: RNAi efficiently downregulates EZH2 expression. (**A**) qPCR (top) and semi-quantitative PCR (bottom) revealed that siEZH2s efficiently depleted EZH2 mRNA expression. $*P < 0.05$. (**B**) Western blotting revealed that siEZH2s remarkably inhibited EZH2 protein expression. $*P < 0.05$. The expression level of EZH2 mRNA was normalized to ACTB for qPCR analysis. Protein expression levels of EZH2 were normalized to GAPDH. The expression levels of EZH2 mRNA and protein were normalized to 1 for the control transfection of siControl. All of the data are plotted as the mean ± SEM from three independent PCR amplifications or western blot assays.

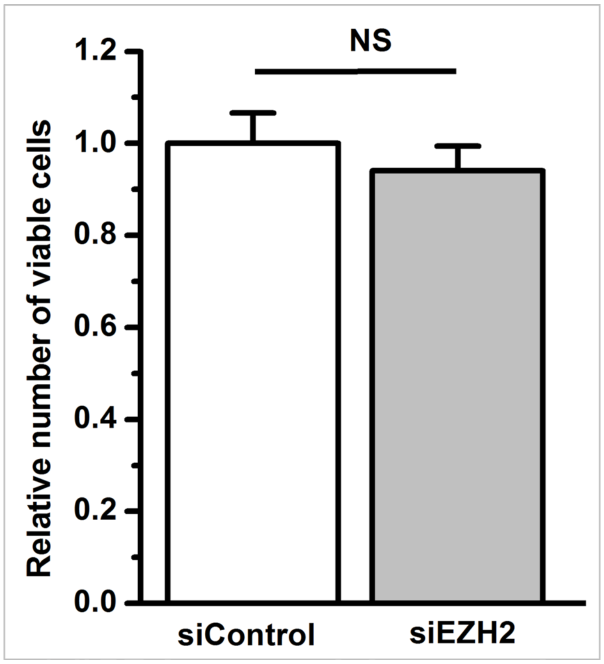

Supplementary Figure S4: RNA interference knockdown of EZH2 did not affect cell proliferation of A549 cells at 9 hours post cell plating. The cell proliferation rates of A549 cells transfected with siControl and those transfected with siEZH2 were similar at 9 hours after cell plating in a 24-well plate. NS, not statistically significant. The number of viable A549 cells transfected with siEZH2 was normalized to 1 for that of viable A549 cells transfected with siControl. All of the data shown represent the mean of at least three independent experiments. The data in all bar graphs are plotted as the mean ± SEM.

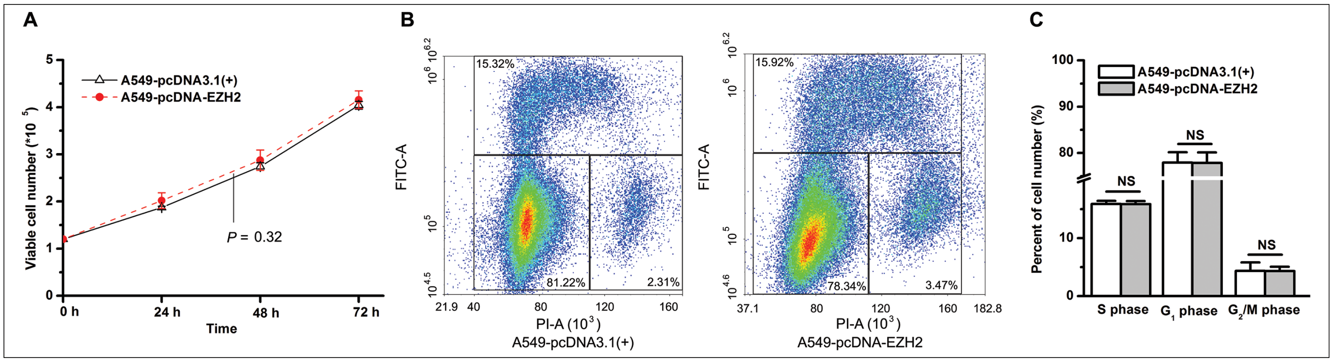

Supplementary Figure S5: Stable overexpression of EZH2 did not change cell proliferation or cell cycle profile. (**A**) The ectopic overexpression of EZH2 in A549 cells did not affect cell proliferation as determined by enumeration of viable cells after cultured for 24 hours, 48 hours and 72 hours. (**B**) Representative cell cycle distribution profiles obtained from A549 cells stably transfected with control plasmid (left) and transfected with *ezh2* plasmid (right). (**C**) Stable overexpression of EZH2 did not change the cell cycle profile. NS, not statistically significant. The cell cycle analysis was performed three times with similar results, and triplicate flasks were included in each experiment. All data shown represent the mean of at least three independent experiments. The data in all bar graphs are plotted as the mean ± SEM.

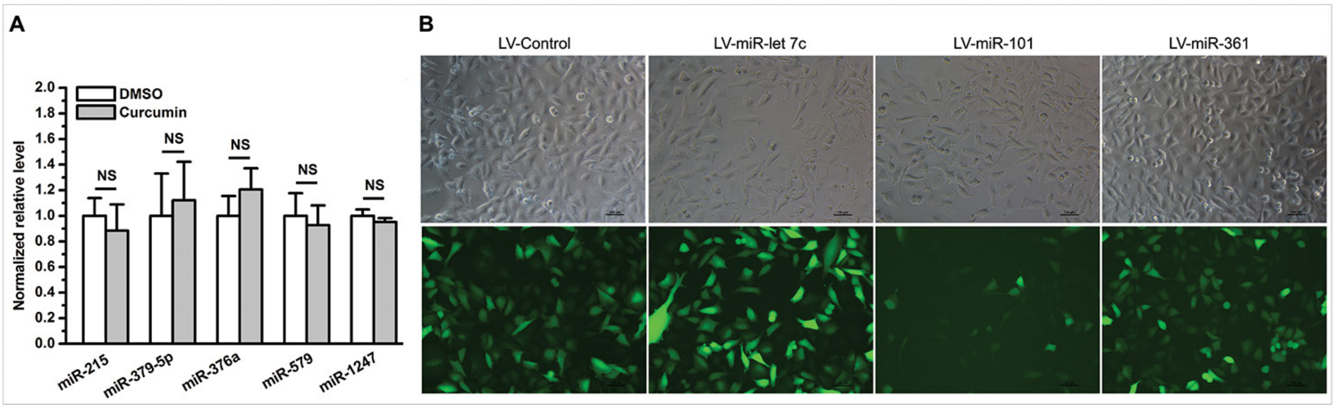

Supplementary Figure S6: Effect of Curcumin on the expression levels of miRNAs and lentivirus transduction of A549 cells. (**A**) qPCR revealed that compared to DMSO, curcumin did not change the expression levels of miR-215, miR-379–5p, miR-376a, miR-579, and miR-1247. NS, not statistically significant. The expression level of each miRNA was normalized to that of the U6 promoter. The expression levels of miRNA were normalized to 1 for the control treatment of DMSO. The data are plotted as the mean ± SEM from three independent PCR amplifications. (**B**) Transduction of A549 cells with lentiviruses expressing enhanced green fluorescent protein (EGFP) (MOI = 15), as observed 72 hours post-transduction under a phase contrast microscope (upper panels) and a fluorescent microscope (bottom panels). Magnification bars = 100 μm.

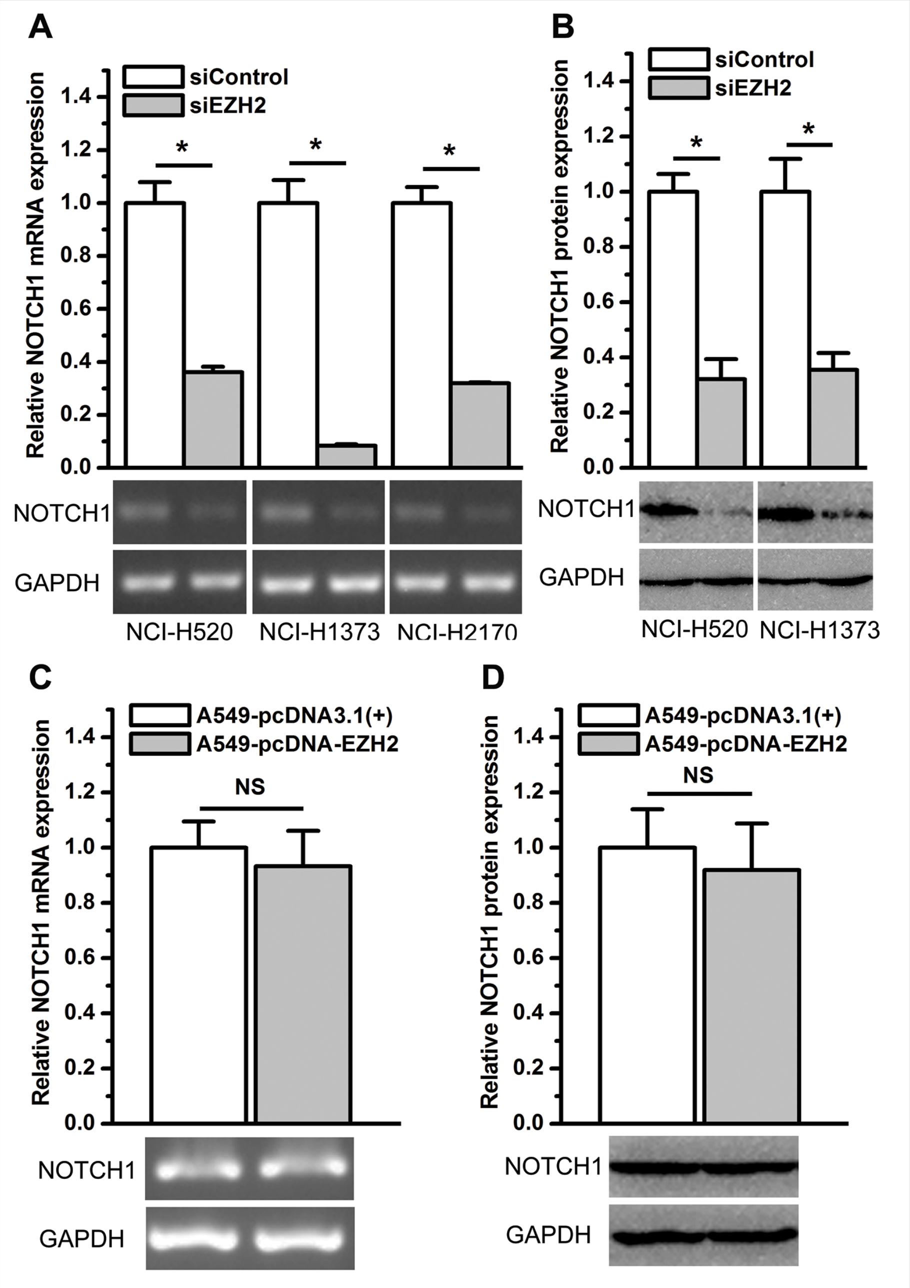

Supplementary Figure S7: EZH2 regulation on NOTCH1 expression. (**A**) Semi-quantitative PCR demonstrated that knockdown of EZH2 inhibited NOTCH1 mRNA expression in the lung cancer cell lines NCI-H520, NCI-H1373 and NCI-H2170. *$P < 0.05$. (**B**) Western blot analysis demonstrated that knockdown of EZH2 inhibited NOTCH1 protein expression in the lung cancer cell lines NCI-H520 and NCI-H1373. *$P < 0.05$. The mRNA and protein expression levels of NOTCH1 were normalized to GAPDH. Expression levels of NOTCH1 mRNA and protein were normalized to 1 for the control transfection of siControl. (**C**) qPCR (top) and semi-quantitative PCR (bottom) demonstrated that ectopic overexpression of EZH2 did not change the expression level of NOTCH1 mRNA. (**D**) Western blot analysis demonstrated that ectopic overexpression of EZH2 did not affect the protein expression level of NOTCH1. NS, not statistically significant. The expression level of NOTCH1 mRNA was normalized to ACTB for qPCR analysis. The protein expression levels of NOTCH1 were normalized to GAPDH. The expression levels of NOTCH1 mRNA and protein were normalized to 1 for the stably transfected control. All data are plotted as the mean ± SEM from three independent PCR amplifications or western blots. The data are plotted as the mean ± SEM from three independent PCR amplifications or western blot assays.

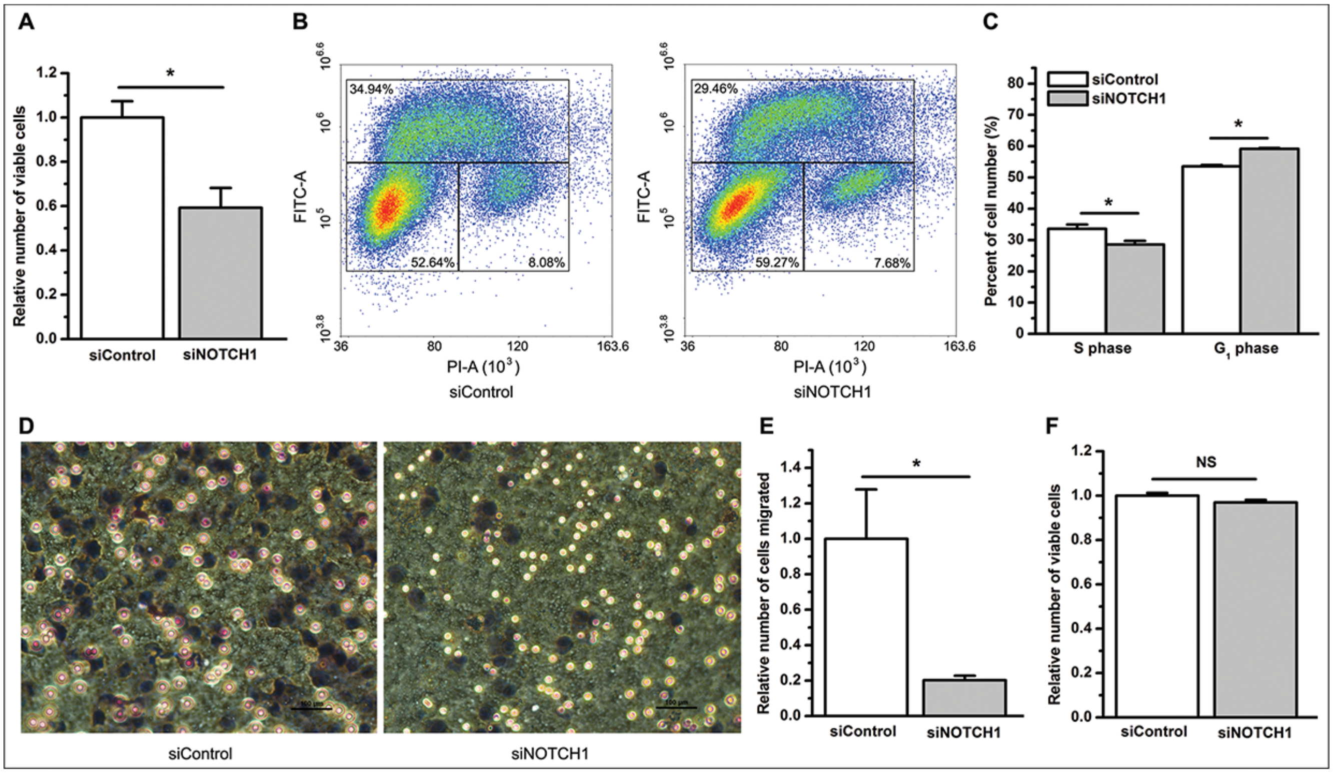

Supplementary Figure S8: Knockdown of NOTCH1 decreased cell proliferation, induced cell cycle arrest, and suppressed cell migration. (**A**) Knockdown of NOTCH1 using siRNA decreased the number of viable cells as determined by the enumeration of viable cells. $*P < 0.05$. The number of viable A549 cells transfected with siNOTCH1 was normalized to 1 for that of viable A549 cells transfected with siControl. (**B**) Representative cell cycle distribution profiles obtained from A549 transfected with siControl (left) and siNOTCH1 (right). (**C**) Knockdown of NOTCH1 using siRNA decreased the percentage of cells in the S phase and increased the percentage of cells in the G_1 phase. $*P < 0.05$. The cell cycle analysis was performed three times with similar results, and triplicate flasks were included in each experiment. (**D**) Compared to A549 cells transfected with siControl (left), transfection of A549 cells with siNOTCH1 inhibited A549 cells from migrating through the membrane of transwell inserts (right). (**E**) The relative number of cells that migrated through the membrane of transwell inserts. $*P < 0.05$. The number of migrated cells in the siNOTCH1 group was normalized to 1 for that of migrated cells in the siControl group. (**F**) The cell proliferation rates of A549 cells transfected with siControl, and those transfected with siNOTCH1 were similar at 9 hours after cell plating in a 24-well plate. NS, not statistically significant. The number of viable A549 cells transfected with siNOTCH1 was normalized to 1 for that of viable A549 cells transfected with siControl. All data shown represent the mean of at least three independent experiments. The data in all bar graphs are plotted as the mean ± SEM.

Supplementary Table S1: Primer sequences for qPCR and semi-quantitative PCR and siRNA sequences

No.	Name		Sequence
1	Primer 1	Forward	5′-CTAGCTAGCTAGATGGGCCAGACTGGGAAGAAATCTGAGAAG-3′
		Reverse	5′-GGGGTACCCCTCACTTGTCGTCATCGTCTTTGTAGTCAGGGATTTCCATTTCTCT-3′
2	Primer 2	Forward	5′-CTAGCTAGCTAGGGGCGACAGTGTGAGACTCCGTTTCTTA-3′
		Reverse	5′- CCCAAGCTTGGGCACTGCCTTCTGAGTCCCACCGGGTGTC -3′
3	EZH2 (qPCR)	Forward	5′-GGACGGCTTCCCAATAACAGTA-3′
		Reverse	5′-CCTGCTTCCCTATCACTGTCTGTA-3′
4	EZH2	Forward	5′-GGCAGCCTTGTGACAGTT-3′
		Reverse	5′-GATGGTGCCAGCAATAGAT-3′
5	NOTCH1 (qPCR)	Forward	5′-TTATGTAGTTGTTCGTTGGTTA-3′
		Reverse	5′-AAAGGGTAGGATGCCTCCGTGT-3′
6	ACTB (qPCR)	Forward	5′-CTCTGGCCGTACCACTGGC-3′
		Reverse	5′-GTGAAGCTGTAGCCGCGC-3′
7	GAPDH	Forward	5′-TGCCTCCTGCACCACCAACT-3′
		Reverse	5′-CCCGTTCAGCTCAGGGATGA-3′
8	siEZH2-a		5′-GGAUGUGGAUACUCCUCCAAGGAAA-3′
9	siEZH2-b		5′-GACCACAGUGUUACCAGCAUUUGGA-3′
10	siEZH2-c		5′-GAGCAAAGCUUACACUCCUUUCAUA-3′
11	siNOTCH1		5′-GGCUGCGGGGUGCUGCUGUCCCGCA-3′

No.1 (Primer 1): primer for the amplification of EZH2 coding sequence from pCMV6-EZH2.
No.2 (Primer 2): primer for the amplification of *EZH2* promoter (–1772 to +112 relative to the start of the first exon).
No.3 (EZH2 (qPCR)): primer for qPCR test of EZH2.
No.4 (EZH2): primer for semi-quantitative PCR test of EZH2.
No.5 (NOTCH1(qPCR)): primer for qPCR test and semi-quantitative PCR test of NOTCH1.
No.6 (ACTB (qPCR)): primer for qPCR test of β-Actin.
No.7 (GAPDH): primer for semi-quantitative PCR test of GAPDH.
No.8 (siEZH2-a): siRNA sequence targeting EZH2 (a).
No.9 (siEZH2-b): siRNA sequence targeting EZH2 (b).
No.10 (siEZH2-c): siRNA sequence targeting EZH2 (c).
No.11 (siNOTCH1): siRNA sequence targeting NOTCH1.
